# Bone-to-Brain: A Round Trip in the Adaptation to Mechanical Stimuli

**DOI:** 10.3389/fphys.2021.623893

**Published:** 2021-04-28

**Authors:** Laura Gerosa, Giovanni Lombardi

**Affiliations:** ^1^Gruppo San Donato Foundation, Milano, Italy; ^2^Laboratory of Experimental Biochemistry & Molecular Biology, IRCCS Istituto Ortopedico Galeazzi, Milano, Italy; ^3^Department of Athletics, Strength and Conditioning, Poznań University of Physical Education, Poznań, Poland

**Keywords:** osteokines, exercise, biomechanical stimulation, mechanosensing, blood-brain barrier, neurodegenerative diseases

## Abstract

Besides the classical ones (support/protection, hematopoiesis, storage for calcium, and phosphate) multiple roles emerged for bone tissue, definitively making it an organ. Particularly, the endocrine function, and in more general terms, the capability to sense and integrate different stimuli and to send signals to other tissues, has highlighted the importance of bone in homeostasis. Bone is highly innervated and hosts all nervous system branches; bone cells are sensitive to most of neurotransmitters, neuropeptides, and neurohormones that directly affect their metabolic activity and sensitivity to mechanical stimuli. Indeed, bone is the principal mechanosensitive organ. Thanks to the mechanosensing resident cells, and particularly osteocytes, mechanical stimulation induces metabolic responses in bone forming (osteoblasts) and bone resorbing (osteoclasts) cells that allow the adaptation of the affected bony segment to the changing environment. Once stimulated, bone cells express and secrete, or liberate from the entrapping matrix, several mediators (osteokines) that induce responses on distant targets. Brain is a target of some of these mediator [e.g., osteocalcin, lipocalin2, sclerostin, Dickkopf-related protein 1 (Dkk1), and fibroblast growth factor 23], as most of them can cross the blood-brain barrier. For others, a role in brain has been hypothesized, but not yet demonstrated. As exercise effectively modifies the release and the circulating levels of these osteokines, it has been hypothesized that some of the beneficial effects of exercise on brain functions may be associated to such a bone-to-brain communication. This hypothesis hides an interesting clinical clue: may well-addressed physical activities support the treatment of neurodegenerative diseases, such as Alzheimer’s and Parkinson’s diseases?

## Introduction

In the last few years, a role for bone tissue in homeostasis has emerged as it solves fundamental functions in the body. Indeed, beyond providing mechanical support and protection to the body and solving important roles in hematopoiesis, calcium storage, ion homeostasis, and phosphate metabolism, additional functions have been described (Calvi et al., 2003; Bergwitz and Juppner, 2010). As a mechanosensitive organ, bone is a dynamic player in biomechanics and body-environment relation and nervous system communication for establishing functional sensing and motor behavior. Indeed, bone and nervous system communicate with each other through a bidirectional connection. Thus, bone emerges as a complex peripheral element able to communicate not only with peripheral organs but also with brain both indirectly, through the peripheral nervous system (PNS), and directly, by releasing molecules able to cross the blood brain barrier (BBB) and to act at the brain level. Noteworthy, recent findings demonstrate that bone acts also as an endocrine tissue, dynamically responsive to internal and external stimuli (Cappariello et al., 2016). It actively communicates with other organs, thus becoming a fundamental key player in the circuit, whose goal is to adapt the body to specific environment. Further, bone, through its endocrine function, regulates whole body homeostasis, energy metabolism, fertility, at least in males, and, as recently established, cognitive functions (Takeda et al., 2002; Yadav et al., 2009; Oury et al., 2011, 2013).

With this review, we want to highlight the importance of the communication between bone and the nervous system, with particular emphasis on the contribution of this relation during mechanical stimulation. In the first part, we will describe the bony elements that act as mechanosensors and the way they respond to stimuli. We than describe how nervous system and bone are connected and communicate with each other to regulate bone homeostasis and bone remodeling depending on biomechanical stimulation it receives. We will first describe how the brain communicate with bone. Finally, although the current little knowledge, we will focus on the bone-to-brain communication based on the new findings on the bone-derived molecules potentially involved in this axis.

A deepen knowledge of the bidirectional communication system between bone and brain is of fundamental interest to address the investigation on the mechanisms underlying bone response and adaption to biomechanical stimuli. It would also help in the search for new targets of proper therapeutic interventions aimed at restoring or ameliorating the adaptive response, especially for those pathologies (e.g., osteoporosis, bone fragility, etc.) that impairs this circuit, but it may also help at improving conditions affecting the nervous system. Thus, our final goal is to bring out the bone and the molecules that it releases as putative therapeutic targets for neurological pathologies that may be also characterized by bone defects, as osteoporosis, bone fragility and increased fracture risk.

## Biomechanical Stimulation and Mechanosensing in Bone

Biomechanical stimulation of bone is fundamental to regulate bone homeostasis, guiding resident cells to adaptation, maintenance, and repair, but also to adapt the entire body to the changing environment.

It is well known that bone is not a static element, but it is able to remodel its cellular components and its entire structure according to the different stimulation it receives, from static or simple movements to the physical activity-generated multidirectional stresses (Duncan and Turner, 1995). There is a finely regulated process that maintain an equilibrium between bone resorption and bone formation that is fundamental for bone homeostasis that is regarded as bone remodeling. This process is mediated by osteoclasts and osteoblasts, which are responsible for bone resorption and for extracellular matrix (ECM) deposition and bone formation, respectively (Parfitt, 2000; Florencio-Silva et al., 2015; Owen and Reilly, 2018). Noteworthy, osteoclasts derive from precursor cells of the monocyte lineage and their differentiation process is mainly stimulated by the activation of receptor activator of nuclear factor κB (RANK) by the RANK ligand (RANKL) expressed and released by stromal cells and osteoblasts, other than immune cells (Suda et al., 1999). On the contrary, osteoprotegerin (OPG), a decoy receptor for RANKL, that is expressed by osteoblasts and osteocytes, interferes with RANKL–RANK binding, thereby inhibiting osteoclasts differentiation and, consequently, bone resorption (Simonet et al., 1997).

The activity of osteoclasts and osteoblasts and, hence, the overall bone remodeling, are regulated by mechanical stimuli (loading and exercises), but also by endocrine and paracrine signals (David et al., 2007; Sen et al., 2011). In this context, external stimuli corresponding to body stationary or moving in the space, converge in the biomechanical stimulation of those bony elements called mechanosensors. These mechanosensors are, for definition, able to sense and respond to biophysical factors in the environment. Indeed, the skeleton must remodel itself to accommodate functional demands (e.g., change in loading intensity and/or direction) (Thompson et al., 2012). In case of chronic intense physical activity (i.e., loading), bones become larger and stronger; on the contrary, a reduction in loading intensity (i.e., bed rest, immobilization) corresponds to enhanced bone resorption that hesitates in reduced bone strength and mass and increased fracture risk.

Bone is a mechanosensitive organ in which at least four elements are considered the mechanosensory elements that collaborate with each other to regulate bone modeling and remodeling. These elements correspond to the main cell types resident into the bone tissue: i.e., osteocytes, osteoblasts, osteoclasts, and osteoprogenitor cells (mesenchymal stem cell, MSC). An important consequence of mechanical stimulation is an indirect regulation of osteoclasts function and of their recruitment through the expression of RANKL by osteoprogenitor cells (Yasuda et al., 1998). Osteoprogenitor cells and pre-osteoblastic cells, located within the bone marrow and in the periosteum, which are mechanically active environment, respond to mechanical stimuli through the regulation of their proliferation, differentiation, and commitment, and, thus, modulating osteoblastogenesis (David et al., 2007; Sen et al., 2011). Further, even if osteocytes are considered the main principal mechanoresponsive elements in bone, it has been demonstrated that also osteoblasts are able to respond to mechanical stimuli (Xiao and Quarles, 2015). Thus, mechanostimuli regulate directly and indirectly all these cells since each of them could respond to mechanical stimulation by modulating pathways that bring to the co-regulation of the other actors.

### Osteocytes as the Main Bone Mechanosensory Elements

Osteocytes are the most abundant cells present in the bone tissue and are considered the main cell types that respond to mechanical stimulation, regulating mechanosensing, and mechanotransduction (Thompson et al., 2012). Further, osteocytes solve two other important roles: they regulate bone homeostasis throughout the regulation of osteoclast and osteoblast activity, and they act as endocrine elements by secreting hormone-like mediators that affect the functioning of cells in bone as well as in other tissues and organs (Chen et al., 2015; Robling and Bonewald, 2020).

These functions are facilitated by their peculiar morphology that allows a direct contacting with other bone cells as well as a direct connection with the interstitial fluid and, hence, with blood. Indeed, these cells are star-shaped cells embedded into the mineralized ECM of bone with prolongations developed within a lacuno-canalicular system (LCS) that put in communication each osteocyte with several other osteocytes and osteoblasts and osteoclasts and also with complex structures such as bone marrow and blood vessels. Further, LCS is filled by an interstitial fluid, that surrounds osteocytes’ body and prolongations that is in equilibrium with the plasma and exposes osteocytes to blood-brought factors derived from distal organs. Thanks to the dynamics governing the fluid movement within the LCS, osteocytes are principally stimulated by shear stress and sense different concentrations of the soluble mediators transported by the fluid (Bonewald, 2017). Osteocytes integrate these inputs and generate chemical signals that coordinate the proper response of the other bone cells. The perception of mechanical stimuli results in the regulation of osteoblast and osteoclast functions thereby influencing bone formation and resorption. Osteocytes may sense mechanical stimuli thanks to multiple mechanosensitive structures, such as cytoskeleton, dendritic processes, integrin-based focal adhesions, connexin-based intercellular junctions, primary cilium, ion channels, and ECM (Dallas et al., 2013; Qin et al., 2020; Robling and Bonewald, 2020).

During mechanotransduction, in osteocytes, the first event that occurs is the increase in intracellular calcium, that derives not only from the external compartment, but also from internal stores, such as those in endoplasmic reticulum (ER) (Lewis et al., 2017). After this first event, several pathways are activated and intervene into the regulation of bone homeostasis.

#### Wnt/β-Catenin

Mechanical stimulation activates the canonical Wnt pathway and influences osteocytes regulation of bone formation during load (Galli et al., 2012; Holguin et al., 2016). Typically, in unloaded state osteocytes secretes inhibitors of the Wnt pathway, namely sclerostin and Dickkopf-related protein 1 (Dkk1), thus favoring osteoclastogenesis. Under loading, instead, the osteocytic expression of sclerostin and Dkk1 is inhibited and, consequently, the Wnt ligands are able to activate this pathway, which, in mature osteoblasts, leads to a direct stimulation of osteoblastogenesis and osteoblast migration, as marked by the expression of the tardive differentiation marker osteopontin (OPN), and inhibition of osteoclastogenesis throughout the release of OPG (Galea et al., 2017). On one hand, the Wnt-induced OPG blocks the RANKL-RANK interaction and inhibits the differentiation of osteoclasts while, on the other hand, OPN prevents bone resorption since it is an activator of osteoblastogenesis with positive role for bone formation in a mechanically stimulated environment (Morinobu et al., 2003).

#### Focal Adhesions

Focal adhesions (FAs) are networks of proteins that dynamically connect the ECM to the intracellular actin cytoskeleton. Thus, extracellular fluid movements are transmitted inside the cells through these membrane proteins anchored to ECM (Chen et al., 2003). Integrins and adhesome proteins are the principal elements that cooperate in focal adhesions leading, in osteocytes, to a mechanosensory response. These protein complexes represent important mechanosensors in osteocytes and regulate skeletal development but also bone mechanobiology (Salter et al., 2001).

#### Apoptosis and Senescence

Appropriate mechanical stimulation prevents osteocytes apoptosis. Mechanical stimulation, indeed, promotes Erk activation supporting osteocytes survival, but, if the loading is too high, it induces damages and, in turn, promotes osteocytes apoptosis (Plotkin et al., 2005; Cardoso et al., 2009). Thus, only an appropriate loading (in terms of intensity and duration) prevents osteocytes senescence and improves their viability.

#### Sclerostin

As reported above, this is an antagonist of Wnt-β catenin signaling that stimulates bone resorption and inhibits bone formation (Li et al., 2005). It has been found high in circulation of subjects during prolonged bed rest and immobilization (Spatz et al., 2015). Thus, a proper mechanostimulation, consequently, to correct exercising, could downregulate the circulating levels of sclerostin, thereby inhibiting bone resorption process.

#### YAP/TAZ

These proteins are two proto-oncogenes that act as mechanosensors and mechanotransducers in different cell types (Dupont et al., 2011). In osteocytes these proteins translocate into the nucleus following mechanical stimulation transduced by both FAs linked with F-actin and by piezo ion channel activated after mechanical stimulation of membrane (Li et al., 2019). This translocation activates several pathways that stimulate bone formation and maintenance of bone mechanical properties, even if the precise mechanism that determines the nuclear translocation of YAP/TAZ is not well defined (Kegelman et al., 2018, 2020).

**FIGURE 1:**
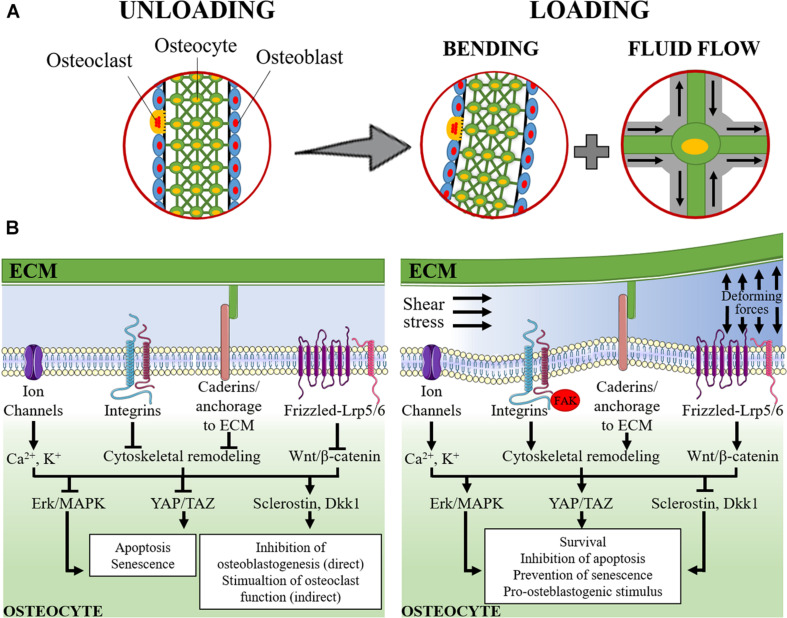
Schematic representation of mechanosensing in osteocytes. **(A)** The lacuno-canalicular system is schematically modeled with osteocytes (green cells) that take contact with adjacent osteocytes, within the bone matrix, and with osteoblasts (light blue cells) and osteoclasts (yellow cells) on the surface of the bone segment. On the left, it is represented the rest status (unloading). On the right it is represented the loading condition: the applied forces cause the bending of the bony segment while, within the lacuno-canalicular system, the interstitial fluid is redistributed and its flow submits osteocytes to shear stress and change in extracellular pressure. **(B)** The signaling pathways activated under unloading (left) and loading (right) conditions are schematically represented. During unloading, pro-apoptotic and pro-senescence pathways are activated (consequently to the inhibition of Erk/MAPK and YAP/TAZ) together with the induction of sclerostin and Dickkopf-related protein 1 (Dkk1). These latter mediators are released into the intercellular fluid and reach osteoblasts and pre-osteoblasts where they exert their anti-osteoblastogenic effects, thereby, indirectly favoring the osteoclast function. During loading, the applied forces and the intra-canalicular fluid shear stress cause the deformation of the osteocyte plasma membrane and of the extracellular matrix (ECM). This results in: (i) perturbation of the electrolyte homeostasis (Ca^2+^ and K^+^); (ii) activation of cadherin and integrin-mediated signaling and the associated focal adhesion kinase (FAK) pathway that result into the remodeling of the cytoskeleton; and (iii) activation of the Wnt/β-catenin signaling. The downstream signaling determines the activation of Erk/MAPK and YAP/TAZ that, together with the inhibition of the expression of sclerostin and Dkk1, result into the stimulation of osteocyte survival, inhibition of apoptosis and prevention of senescence and, in turn, a support to osteoblastogenesis and osteoblast function.

A schematic representation of the mechanosensing and mechanosensory pathways in osteocyte are represented in Figure 1.

### Bidirectional Connections Between Brain and Bone

In the first part of this review, we have briefly illustrated the complex mechanosensing strategies occurring in bone and how much dynamic is this organ in order to actively respond to biomechanical stimuli and, consequently, to allow the body to adapt to the changing environment. Further, we will go deeper into the complexity of bone mechanosensing, highlighting the existing relationship with the nervous system. Indeed, besides the direct regulation of bone metabolism, homeostasis and remodeling that occur at the bone cell level, depending on the received mechanical stimuli received from bone fluid movements, skeletal muscle traction and ground/impact reactions, another important mechanism controlling bone response and metabolism involves the nervous system.

Bone can be regulated both directly by PNS and indirectly by central nervous system (CNS). However, it has recently emerged a strong two-way interaction between bone and brain, that could be defined as bone-brain axis: not only brain regulates bone (efferent pathway), but also bone can communicate to the brain (afferent pathway) through the release of molecules, i.e., osteokines, that can act in the brain and, indeed, have been found in brain (Brazill et al., 2019; Millar et al., 2019).

In addition to the soluble mediators, other routes of inter-organ cross-talk exist. An intriguing, and increasingly studied, path is represented by the extracellular vesicles (EVs) and their cargo. EVs are cell-derived membrane vesicles, differing in size, biogenesis and membrane protein profile [i.e., exosomes, microvesicles (MVs), apoptotic bodies]. Exosomes and MVs are involved in paracrine and endocrine intercellular communication. They are actively released from the source cell and selectively loaded with specific components sorted from the cytosol, to reach a target cell in which the vesicle content elicits a biological response. EVs mediate the direct transfer of the contained information into the target cell and the EV-mediated information transfer is implicated in several diseases (Faraldi et al., 2020). There are evidence about an EV-mediated crosstalk between brain and bone, although this field still needs to be fully depicted.

We will firstly describe how nervous system can communicate with bone, mechanisms whose functioning are well known, to then move to the more innovative bone-to-brain view that, however, still needs deeper investigations.

## Brain-To-Bone Communication

### Direct Regulation of Bone by the Peripheral Nervous System: Bone Innervation

The autonomous nervous system is known to regulate the peripheral functions prompt to maintain body homeostasis and to initiate the adaptive response to various stress, including the biomechanical stimulation. It acts through two antagonistic system: the parasympathetic nervous system, which favors the “rest and digest” response; and the sympathetic nervous system, that is responsible for the “fight or flight” response.

Noteworthy, the parasympathetic nervous system acts through the release of acetylcholine (ACh), a neurotransmitter that activates both muscarinic and nicotinic cholinergic receptors, while sympathetic nerves release norepinephrine (NE), a neurotransmitter that acts via α- and β- adrenergic receptors (α-AR and β-AR) expressed by pre-synaptic and post-synaptic terminals, respectively.

During the last years, the development of new techniques led to the demonstration that bone is a highly innervated organ from both sympathetic and sensory neurons, thus showing that bone is physically related to the PNS (Mach et al., 2002). Interestingly, histological analyses showed high densities of nerves, belonging to the autonomous branch, in areas of high osteogenic activity. Further, immunocytochemistry experiments have successfully demonstrated the presence of receptors for neural peptides on bone cells (Elefteriou, 2005). Finally, the importance of the brain-bone connection has been further supported by the identification of neural tracts between femoral bone marrow and the CNS using retrograde trans-synaptic signaling (Denes et al., 2005).

#### Parasympathetic Innervation of Bone

Nicotinic acetylcholine receptor (nAChR) subunits are expressed by osteoclasts and differentiating and mature osteoblasts (Mandl et al., 2016). Further, transcript of muscarinic AChR type M1, M2, and M4 have been found in immature and differentiated osteoblasts (Sato et al., 2010). Transcripts of both AChR types have been detected in murine osteocyte-like MLO-Y4 and their levels are modulated by ACh, but the precise pathways activated by ACh in these cells are still unknown (Ma et al., 2014). Besides AChRs, osteoblasts express the machinery to synthesize ACh, but the functional role of this presence remains unknown (En-Nosse et al., 2009). The expression of both nicotinic and muscarinic ACh receptors on osteoblasts, osteocytes, and osteoclasts suggests that these cells are directly regulated by the parasympathetic nervous system. It has been observed both *in vitro* and *in vivo* experiments that activation of nAChR inhibits RANKL-dependent osteoclastogenesis, even if more experiments are needed to better elucidate the specific role on bone homeostasis of the different subunits of nAChRs since some results are contradictory (Mandl et al., 2016). Further, it has been observed that agonists of nAChR increase osteoclasts apoptosis and restrain bone resorption (Bajayo et al., 2012). All these evidences suggest that parasympathetic nervous system inhibits bone resorption and, thus, promotes bone formation (Table 1).

**TABLE 1:** Peripheral nervous system to bone communication.

Nervous system branch	Neurotransmitter	Receptor	Target cell	Main action
Parasympathetic	ACh	nAChR	Osteoblast	Inhibition of
		mAChR	Osteoclast	bone resorption
Sympathetic	NE	αAR	Osteoblast	Promotion of
		βAR	Osteoclast	bone resorption
Sensory	CgRP	CgRPR	Osteoblast	Promotion of bone formation
	SP	SPR	Osteoclast	
	Sem3A	Nrp1, Plxna1, 2, 3		

*Ach, acetylcholine; nAChR, nicotinic acetylcholine receptor; mAChR, muscarinic acetylcholine receptor; NE, norepinephrine; αAR, alpha adrenergic receptor; βAR, beta adrenergic receptor; CgRP, calcitonin gene-related peptide; SP, substance P; Sem3A, semaphorin 3A; CgRPR, calcitonin gene-related peptide receptor; SPR, substance P receptor; Nrp1, sema3A receptor; Plxnal1, 2, 3, sema3A co-receptors.*

#### Sympathetic Innervation of Bone

Sympathetic action on bone remodeling could be hypothesized after the observation of very low levels of mRNA of α-ARs in osteoblasts and osteoclasts and higher levels of β-ARs. Consequently, β-ARs may be the main AR to mediate the action of sympathetic nerves in bone (Khosla et al., 2018). Further, β-ARs have been also found in osteocyte-like MLO-Y4 cells (Yao et al., 2017). Osteoblasts and osteoclasts express the β-2AR, but the biological relevance of the action of these receptors on osteoclasts is still not known. What is known is that the stimulation of β-2AR leads to increased osteoclasts formation, impairs osteoblasts functions and, consequently, increases bone loss. The inhibition of these receptors, on the contrary, leads to enhanced bone formation (Elefteriou, 2008). Moreover, stimulation of β-ARs in osteocyte-like MLO-Y4 cells affects osteoclastogenesis by increasing the RANKL-to-OPG ratio (Yao et al., 2017; Liang et al., 2018).

In summary, the regulation of bone remodeling by the autonomous nervous system results in the promotion of bone formation by the parasympathetic system and, conversely, in favoring bone resorption by sympathetic nervous system (Table 1).

#### Sensory Innervation of Bone

Besides the parasympathetic and sympathetic activities in bone, it has been shown that sensory nerves are important for bone formation and solve fundamental roles in their response to mechanical loading. The sensory innervation represents the third arm of the autonomous system involved in the regulation of bone remodeling. The existence of such innervation in bone has been demonstrated through the detection of sensory fibers in bone and also of calcitonin gene-related peptide (CGRP) and substance P (SP), which are neuropeptide released by sensory fibers and acting as neurotransmitters (Mach et al., 2002). The receptors for these neuropeptides have also been detected in osteoblasts and osteoclasts (Kodama et al., 2017). Low amounts of NK1-R, a SP receptor, have been detected in osteocytes, however, the specific function in these cells is still unknown (Goto et al., 1998). CGRP and SP stimulate proliferation and activity of osteoblasts, thus influencing bone formation. Specifically, CGRP stimulates bone formation induced by mechanical loading (Ma et al., 2013; Sample et al., 2014). A role for the sensory nervous system in bone remodeling, as promoter for bone formation, has been demonstrated, too, through denervation studies and investigation on the bidirectional communication between sensory neurons and osteoblasts in co-culture systems (Ding et al., 2010; Kodama et al., 2017). In particular, co-culture experiments lead to the observation that sensory neurons release glutamate and SP that act on osteoblasts, while osteoblasts release ATP that acts on sensory neurons (Kodama et al., 2017).

Other important neuropeptides, known to regulate bone homeostasis, are semaphorins. Particularly, sema3A, released by sensory fibers, plays a role in the regulation of bone remodeling, by promoting bone mass gain and dendritic osteocyte elongation, by acting on Nrp1 receptor and Plxna1, 2, 3 co-receptors. Indeed, it has been shown that mice knockout for sema3A experienced a decrease in bone mass and, consequently, to a reduction in bone formation (Fukuda et al., 2013; Niimura et al., 2016) (Table 1). Further, sema3A deficiency in osteocytes leads to severe osteopenia, in aged mice, since it promotes osteocyte survival (Hayashi et al., 2019).

### Indirect Regulation of Bone by Central Nervous System: Neuroendocrine Signaling

To date, it is known that bone metabolism and remodeling are regulated not only by the PNS but also by CNS through the action of several molecules as neurohormones, neuropeptides and neurotransmitters.

#### Neurohormones That Regulate Bone Metabolism

The principal mechanism of regulation of the peripheral functions by the CNS is mediated through the release of neurohormones by the hypothalamus that stimulate hormone release from the pituitary gland. Osteoblasts and osteoclasts, but not osteocytes, express different receptors for these hormones and it has been demonstrated that some of these regulate skeletal integrity favoring either bone resorption or bone formation (Table 2).

**TABLE 2:** Brain to bone communication: neurohormones.

Neurohormone	Target cell in bone	Main action
FSH	Osteoclast	Stimulation of osteoclastogenesis and osteoclast function
	Osteoclast precursors	
TSH	Osteoblast	Independent regulation of bone formation and bone resorption
	Osteoclast	
Prolactin	Osteoblast	Inhibition of osteoblast proliferation and bone mineralization
ACTH	Osteoblast	Promotion of osteoblast proliferation
GH	Osteoblast	Promotion of bone formation
AVP/ADH	Osteoblast	Inhibition of osteoblastogenesis
	Osteoclast	Stimulation of osteoclastogenesis
OT	Osteoblast	Stimulation of osteoblastogenesis
	Osteoclast	Inhibition of osteoclast activity
Melatonin	Osteoblast	Promotion of osteoblasts differentiation
	Osteoclast	Promotion of osteogenesis

*FSH, follicle-stimulating hormone; TSH, thyroid-stimulating hormone; ACTH, adrenocorticotropic hormone; GH, growth hormone; AVP/ADH, arginine-vasopressin/antidiuretic hormone; OT, oxytocin.*

Both the follicle-stimulating hormone (FSH) and the thyroid-stimulating hormone (TSH) regulate directly bone remodeling. *In vitro* and *in vivo* experiments showed that FSH stimulates formation and function of osteoclasts, promoting bone resorption, by acting through a FSH receptor expressed on the plasma membrane of osteoclasts and their precursors (Sun et al., 2006; Robinson et al., 2010). On the contrary, TSH sustains bone integrity by stimulating osteoblasts functioning and inhibiting osteoclasts activity by acting directly through the TSH receptors expressed by these cells (Abe et al., 2003; Baliram et al., 2013). On one hand, TSH limits bone loss by decreasing osteoclastogenesis and, on the other hand, it restores bone mass by promoting osteoblastogenesis. Further, TSH can suppress osteoblasts differentiation. These pleiotropic actions define TSH as a single and independent molecule that regulate bone remodeling acting on both bone formation and bone resorption (Abe et al., 2003; Sampath et al., 2007; Baliram et al., 2011).

The expression of prolactin receptors has been detected in osteoblasts, but not in osteoclasts, and it has been demonstrated that prolactin contributes to the regulation of bone homeostasis by inhibiting osteoblastic proliferation and bone mineralization (Seriwatanachai et al., 2008, 2009). The indirect prolactin-dependent promotion of bone resorption may be responsible for the mobilization of calcium from bone to be used for milk secretion during lactation.

Adrenocorticotrophic hormone (ACTH) binds to melanocortin receptor family 2 (MC2R) that is expressed by osteoblastic cells and its expression is high at sites of active bone deposition, thus suggesting a role in the promotion of bone formation through the stimulation of osteoblasts proliferation (Zhong et al., 2005; Tourkova et al., 2017).

The growth hormone (GH) stimulates bone gain both indirectly, by stimulating insulin-like growth factors (IGFs) that regulates skeletal development, and directly, by acting on bone cells (DiGirolamo et al., 2007; Dobie et al., 2014).

Arginine-vasopressin (AVP, also known as antidiuretic hormone, ADH) and oxytocin (OT) regulate bone metabolism by acting in opposite ways: AVP impairs osteoblastogenesis and induces osteoclastogenesis by directly acting on AVP receptors expressed in both osteoblasts and osteoclasts; on the contrary, OT promotes osteoblastogenesis and inhibits osteoclast activity by acting on OT receptors expressed in osteoblasts and osteoclasts (Tamma et al., 2013; Sun et al., 2016).

Finally, the expression of the melatonin receptors have been observed in both osteoblasts and osteoclasts and it has been demonstrated that melatonin regulates bone homeostasis by promoting osteoblast differentiation and osteoblastogenesis (Roth et al., 1999; Zhang et al., 2010). Defective melatonin signaling has been associated with impaired osteoblast function and development of scoliosis (Akoume et al., 2019).

#### Neuropeptides That Regulate Bone Metabolism

Bone homeostasis and remodeling are also under the direct control of several neuropeptides released by hypothalamus (Table 3).

**TABLE 3:** Brain to bone communication: neuropeptides.

Neuropeptide	Target cell in bone	Main action
NPY	Osteoblast (Y1 receptor)	Inhibition of osteoblasts function (Y1)
	Hypothalamus (Y2 receptor)	Anti-osteogenic effects (Y2)
AgRP	Osteoblast and osteoclast (throughout the sympathetic nervous system)	Stimulation of osteoblasts activity
CART	No evidences for direct role on bone cells	Stimulation of bone mass gain
Melanocortin	Hypothalamus (MC4R)	Stimulation bone formation
	Osteoblast (MC4R)	
	Osteoblast and osteoclast (MCRs)	
Neuromedin U	Central action–mediated by leptin	Inhibition of bone mass gain
VIP	Osteoblast	Anti-resorptive effect
	Osteoclast	

*NPY, neuropetide Y; AgRP, agouti-related protein; CART, cocaine/amphetamine-related transcript; MC4R, melanocortin 4 receptor; MCRs, melanocortin receptors; VIP, vasoactive intestinal peptide.*

Neuropeptide Y (NPY) has been demonstrated to play important peripheral roles. It is produced centrally in the arcuate nucleus (ARC) of the hypothalamus and regulates bone homeostasis with site-specific effects in periphery (Baldock et al., 2009). Both NPY and the relative Y1 receptors have been found in cells of the osteoblastic lineage. Peripherally NPY exerts catabolic effects in bone through the inhibition of osteoblasts activity and interacts with mechanical signals to integrate the osteoblasts regulation with the local environmental loading status. Specifically, this interconnection and local effects of NPY are mediated by osteocytes which produces local NPY that affects osteoblast activity after mechanical stimuli (Igwe et al., 2009). Noteworthy, the actions of this neuropeptide in bone homeostasis are exerted not only peripherally on Y1 receptors expressed by osteoblasts, but also throughout a central signaling, on CNS throughout the Y2 receptors (Shi et al., 2011; Lee N. J. et al., 2015). In particular, it has been observed that, when activated, Y2 receptors, present in hypothalamic NPY-expressing neurons, elicit anti-osteogenic effects on trabecular but not on cortical bone (Shi et al., 2010). Interestingly, pre-osteocytes and osteocytes express NPY, as demonstrated in neonatal calvaria, and its expression was reduced in response to fluid shear stress. The treatment of calvaria osteoblasts with NPY decreased the intracellular levels of cyclic AMP (cAMP) and limits the expression of the markers of osteoblast differentiation (e.g., osteocalcin (OCN), bone sialoproteins, and dentin matrix acidic phosphoprotein 1-DMP1) (Igwe et al., 2009).

Agouti-related peptide (AgRP) acts through the sympathetic nervous system on bone metabolism. Increased neuronal AgRP activity downregulates the sympathetic tone favoring bone mass gain throughout the enhancement of the osteoblast activity (Kim et al., 2015; Shi et al., 2017).

Cocaine amphetamine regulated transcript (CART) is a neuropeptide precursor protein highly expressed in the hypothalamus, but also peripherally. It has been observed that while low hypothalamic CART expression is associated with increased bone resorption, through the induction of higher levels of RANKL, elevated CART expression resulted an increase in bone mass (Elefteriou et al., 2005).

The melanocortin peptides bind to five different G protein-coupled receptors and they sustain osteo-positive effects by binding to melanocortin 4 receptor (MC4R), which is highly expressed in the hypothalamus (Farooqi et al., 2000). Melanocortin receptor MC4R have been found in mouse periosteum and rat osteoblasts suggesting a direct role for melanocortin in bone development and metabolism (Dumont et al., 2005). Further, other melanocortin receptors have been detected in both osteoblasts and osteoclasts (Zhong et al., 2005). Melanocortin agonists stimulate osteoblast proliferation in *in vitro* models. Further, knock out mice for MC4R experience increase bone mass due to reduced osteoclasts number (Elefteriou, 2005), suggesting that melanocortin promotes bone formation throughout the regulation of the proliferation rate of both osteoblasts and osteoclasts.

Another neuropeptide that regulates bone mass is neuromedin U that elicits bone resorption through a leptin-mediated pathway, acting preferentially at the CNS level rather than peripherally (Sato et al., 2007).

The vasoactive intestinal peptide (VIP) acts through sympathetic and parasympathetic nerve fibers. It is frequently released together with ACh by parasympathetic terminals at the bony periosteum level and, mainly, in the epiphysis. It acts directly on osteoblasts and osteoclasts by binding to its G protein-coupled receptors and exerts an anti-resorptive effect (Togari et al., 1997). It has been shown that VIP inhibits RANKL expression in osteoblasts, while it suppresses RANK in osteoclasts, and, in parallel, induces OPG expression in osteoblast (Mukohyama et al., 2000; Juarranz et al., 2005).

#### Neurotransmitters That Regulate Bone Metabolism

Bone homeostasis and bone remodeling are regulated also by neurotransmitters released by CNS, such as serotonin, glutamate, and dopamine (Table 4). Indeed, bone cells express receptors for these neurotransmitters. Osteoblasts, osteocytes, and osteoclasts express different subtypes of G protein-coupled receptors for serotonin. Particularly, osteocyte-like MLO-Y4 cells express the serotonin receptors 5-HT1A and 5-HT2A, but also the serotonin transporter (5-HTT) and the enzyme involved in serotonin synthesis, thus being an important component of the serotonin system in bone (Bliziotes et al., 2006). Interestingly, the precise mechanism of action of serotonin in regulating bone cells activity is still under investigation and it is supposed that it acts differently depending on its origin: gut-derived serotonin decreases osteoblast proliferation, while serotonin derived from CNS enhances bone formation (Cui et al., 2011; Park et al., 2018).

**TABLE 4:** Brain to bone communication: neurotransmitters.

Neurotransmitter	Receptor	Target cell in bone	Main action
Serotonin	GPCRs for serotonin	Osteoblast	Central serotonin: enhancement of bone formation
		Osteoclast	Peripheral serotonin: inhibition of osteoblast proliferation
Glutamate	NMDA receptors	Osteoblast	Stimulation of osteoblasts differentiation and function
		Osteoclast	Inhibition of osteoclasts activity
Dopamine	DR-1, DR-2, DR-3, and DR-5	Osteoblast	Stimulation of bone formation and mineralization
		Osteoclast	Inhibition of osteoclastogenesis

*GPCRs, G protein-coupled receptors; NMDA, *n*-methyl-D-aspartate; DR, dopamine receptor.*

Osteoblasts and osteoclasts express glutamate receptors, in particular the *N*-methyl-D-aspartate (NMDA) type is quantitatively the most represented (Chenu et al., 1998; Gu et al., 2002). The role of glutamate in bone is interesting and highly complex, since the active glutamate derives both centrally and peripherally and its action involves different cells. On one hand, there is glutamate innervation at the bone levels near bone cells expressing glutamate receptors (i.e., osteoblasts and osteoclasts) but, on the other hand, the entire osteoblasts population expresses the set of components for a regulated release of glutamate (Hinoi et al., 2002; Bhangu, 2003). However, different researches have demonstrated that glutamate inhibits osteoclasts activity and promotes osteoblasts functions (Taylor, 2002; Morimoto et al., 2006). Noteworthy, both the glutamate transporter, GLAST, and the glutamate receptors respond to mechanical loading. *In vivo* studies demonstrated that mechanical load regulates the expression of GLAST and glutamate receptors in bone (Mason et al., 1997; Ho et al., 2005). Interestingly, it has been hypothesized that osteocytes are the first responders to mechanical load in bone. Indeed, after some days of mechanical loading, GLAST protein was undetectable in osteocytes, while its expression increases in osteoblasts (Mason et al., 1997).

Dopamine is another important central neurotransmitter that also affects bone homeostasis. It acts through dopamine receptor (DR)-1, -2, -3, and -5 and enhances osteoblastic proliferation and bone mineralization and suppresses osteoclastogenesis (Hanami et al., 2013; Lee D. J. et al., 2015; Wang et al., 2020). Modulation of dopamine levels as therapeutic interventions for those pathologies featured by a dysregulation of dopamine levels [e.g., Alzheimer disease (AD), Parkinson disease (PD)] could interferes with bone mass (Chen et al., 2016).

### Adipose Tissue and Skeletal Muscles As Two Intermediates in Bone-Brain Axis

Beside a direct brain-to-bone axis, these two organs may communicate throughout an indirect crosstalk involving mediators expressed and released by a third tissue. Adipose tissue and skeletal muscles are, actually, two important organs able to integrate stimuli of different nature (biomechanical and biochemical) and to release mediators (e.g., myokines and adipokines) having effects all over the body and, hence, also in bone either directly or throughout the mediation of the nervous system.

#### Hormones Released by Adipocytes: Adipokines

Actually, among several adipokines, there are two main hormones, released by adipocytes, that act on CNS to regulate bone metabolism and remodeling.

Leptin is released by adipocytes in response to insulin stimulation and glucose uptake, which correspond to anabolic state (Barr et al., 1997; Mueller et al., 1998) and binds receptors located in the CNS involved in the regulation of appetite and energy metabolism. In addition, it binds on receptors located at the hypothalamic level that regulate bone metabolism through the activation of the SNS. Hence, leptin-regulated neural pathways control both aspects of bone remodeling (Takeda et al., 2002; Elefteriou et al., 2005).

Adiponectin also regulates bone metabolism by acting on neurons of locus coeruleus, decreases the sympathetic tone and, therefore, increases bone mass (Kajimura et al., 2013). It is important to note that OCN, a bone-derived hormone whose functions will be discussed below, stimulates the release of adiponectin by adipocytes (Hill et al., 2014; Otani et al., 2015). Thus, it could be considered the existence of an indirect way by which bone, acting through the peripheral adipose tissues and then, through the CNS, regulates its own remodeling.

#### Hormones Released by Skeletal Muscle Cells: Myokines

It is known that skeletal muscles, during their activity (i.e., contraction), release molecules, the so called myokines, that act on other tissues, with a hormone-like activity, to mediate adaptive responses (Gomarasca et al., 2020).

Irisin, one of the more recently identified myokines, is a circulating hormone-like mediator that is released by skeletal muscles during and after exercises (Bostrom et al., 2012; Wrann et al., 2013; Lombardi et al., 2016). It regulates energy metabolism, by stimulating the usage of energy substrates to release heat, and acts not only on skeletal muscles, but also on adipose tissue, bone, and brain. In bone, irisin mediates anabolic effects and acts by stimulating osteoblasts activity and reducing the number of osteoclasts (Colaianni et al., 2015), thus promoting bone formation. If, on one hand, its circulating levels are increased by physical exercise, on the other hand, it is decreased in bone metabolic dysfunction, as in osteoporosis (Anastasilakis et al., 2014). In brain, irisin is expressed in Purkinje neurons of the rat and mouse cerebellum and it is necessary for the proper neural differentiation of embryonic stem cells (Forouzanfar et al., 2015). Further, it may have neuroprotective effects after ischemic stroke and, by acting on hippocampus, it may rescue synaptic plasticity and memory impairment in AD (Asadi et al., 2018; Lourenco et al., 2019; Martinez Munoz et al., 2018). Irisin could be an interesting therapeutic target in osteoporotic traits associated to neurodegenerative disorders, based on its pro-anabolic effects, and in view of the fact that its circulating levels could be increased by physical exercise. However, it is not yet known if peripheral irisin, released by the skeletal muscle, can act directly on CNS, a condition that implies the crossing of the BBB, where it may regulate, among the other effects, bone remodeling after exercise.

## Bone-To-Brain Communication

It has been shown that osteocytes release EVs, throughout a calcium-dependent mechanism, and that these EVs contain, among the others, RANKL, OPG, and sclerostin. This mechanism is supposed to represent a way by which the osteocyte response to mechanical loading may be transmitted to other tissues/organs, as demonstrated by *ex vivo* studies of intact bone that showed an enhanced release of EVs following mechanical stimulation (Morrell et al., 2018). However, very little is known about the central role of the bone-derived mediators. For instance, the precise mechanism by which bone-derived mediators may cross the BBB is not well known and it is still under investigation.

It has been shown that low bone density (BMD) and osteoporosis may associate with dementia and AD in postmenopausal women (Tan et al., 2005; Loskutova et al., 2009). Further, low BMD and BMD loss are risk factors for osteoporosis and AD and is an early risk factor for dementia. Women with high levels of hip bone loss have an increased probability to develop cognitive dementia compared to women with limited loss. In addition, looking at this point from another point of view, it has been shown that high lean body mass is associated with lower risk of cognitive impairment or dementia (Burns et al., 2010; Friedman et al., 2010; Basgoz et al., 2020).

The serum level of several bone-derived mediators has been found modulated in patient affected by bone diseases, as osteoporosis, but also in the case of neurodegenerative diseases, such as AD [e.g., OCN, OPN, sclerostin (Yuan et al., 2019), Dkk-1 (Huang et al., 2018), and lipocalin 2 (Song and Kim, 2018)] and PD [e.g., bone morphogenic protein 2 (Goulding et al., 2020), OCN (Shan et al., 2019)]. Interestingly, in AD, PD, as well as in other neurodegenerative diseases, osteopenia and osteoporosis are often comorbidities (Roos, 2014; Binks and Dobson, 2016). Thus, these molecules might be considered as relevant therapeutic targets in pathological conditions affecting the CNS featured by bone dysmetabolism. Indeed, by acting peripherally, with addressed exercise programs, it may be possible to modulate the levels of these proteins with potential beneficial effects on CNS, other than on bone (Lee et al., 2019).

In this last part of the review, we will discuss the current knowledge around the established and the putative actions of bone-derived molecules in the nervous system, highlighting how their exercise-induced modulation may potentially benefit both organs (Table 5).

**TABLE 5:** Bone to brain communication: hormones and peptides.

Bone-derived mediator	Cells source in bone and brain	Exercise-related modifications	Main actions	
OCN	Osteoblast	↑ Circulating levels by aerobic exercises (mice and human)	BONE	cOCN binds to hydroxyapatite in bone ECM and regulates mineralization
		↑ ucOC by endurance non-weight bearing activities		Marker of bone formation
		↑ cOC by endurance weight bearing/impact activities		
			BRAIN	*Mouse*
				Able to cross the BBB
				Regulation of neurotransmitters synthesis in different brain areas
				Necessary for both brain development and functioning
				Regulation of learning, memory, and cognitive functions
				*Human*
				Correlation between plasma level of OCN and global cognition in women
				Low level of OCN correlates with brain microstructural changes
				The OCN form active in human brain is unknown
LCN2	Osteoblast (at least the 50% of circulating LCN2)	↓ by physical exercises	BONE	Promotion of osteoclastogenesis
		↑ by bed resting		Inverse correlation between LCN2 and BMD, in women
			BRAIN	*Mouse*
				Able to cross the BBB
				Act on hypothalamic MC4R to regulate appetite response
				Increased during metabolic inflammation and acts on hippocampus to modulate inflammation
sclerostin	Osteocyte	↓ by mechanical loading	BONE	Inhibition of osteoblastogenesis through inhibition of Wnt/β-catenin pathway and stimulation of RANKL release
		↑ by bed resting/immobilization	BRAIN	Potential regulation of neuronal function, through Wnt/β-catenin, possibly in neurodegenerative diseases
				Not known if able to cross the BBB
Dkk1	Osteoblast, osteocyte	↓ by chronic exercise (serum levels)	BONE	Inhibition of osteoblastogenesis through inhibition of Wnt/β-catenin pathway
			BRAIN	*Mouse*
				Upregulated in transgenic AD mice
				Contributes to impairment in LTP, learning and memory
				*Human*
				Upregulated in AD patients
				Circulating Dkk1 inversely correlates with cognitive performances in elderly women
				Not known if able to cross the BBB
FGF23	Osteocyte, Osteoblasts, expressed in specific brain areas (hypothalamus, hippocampus, cortex and in cerebrospinal fluid)	↑ in unloading	BONE	Inhibition of osteoblastic activity
				Stimulation of osteoclast-mediated bone resorption
			BRAIN	Not known if able to cross the BBB (evidence for other FGF proteins)
				KO mice have impairment hippocampal cognitive functions
				Not yet well known the role in brain
OPN	Osteoblast	↓ by acute exercise in weight loss program	BONE	Stimulation of bone resorption and demineralization
				Negative correlation with BMD
			BRAIN	Highly expressed in brain sections of PD patients
				High serum level in AD patients
				Regulation of reparative processes in neurodegenerative disorders
RANKL	Osteoblast, immune cells	↓ by acute exercise	BONE	Stimulation of osteoclastogenesis and osteoclast function
			BRAIN	Highly expressed in hypothalamus and involved in the regulation of body temperature and fever; involvement in depressive disorders
BMPs	Osteoblast (synthesis), Osteoclasts (resorption dependent release from ECM)	↑ acute exercise	BONE	Stimulation of bone formation
				Modulation of osteogenesis following mechanical stimulation
			BRAIN	Involved in neurogenesis during embryonic phase and in adulthood
BDNF	CNS, PNS, osteoblasts, and chondrocytes	↑ acute exercise	BONE	Beneficial effects on bone cells, in particular promotion of proliferation and differentiation of MSC into osteoblasts
				Conditional KO in brain mice develops bone defects
			BRAIN	Support to synaptic plasticity, neurodevelopment, and neuronal differentiation
				Reduced circulating levels in AD and PD patients
IGF-1	Osteoblasts, chondrocytes In brain both during embryogenesis and in adulthood (cerebellum, olfactory bulb, hippocampus)	↑ by acute exercise	BONE	Stimulation of bone formation through stimulation of chondrocytes proliferation and osteoblasts differentiation
		↓ by chronic exercises		Decreased serum levels with age and in patients affected by osteoporosis
			BRAIN	Involvement in neurodevelopment and plasticity
				Involvement in aging-associated neurodegenerative disorders

*OCN, osteocalcin; LCN2, lipocalin2; Dkk1, dickkopf-related protein 1; LTP, long term potentiation; FGF23, fibroblast growth factor 23; KO, knockout; OPN, osteopontin; BMD, bone mineral density; RANKL, receptor activator of nuclear factor κB ligand; BMPs, bone-morphogenic proteins; ECM, extracellular matrix; BDNF, brain-derived neurotrophic factor; CNS, central nervous system; PNS, peripheral nervous system; IGF-1, insulin-like growth factor 1.*

### Osteocalcin

Osteocalcin, also known as bone γ-carboxyglutamic acid protein (BGLAP), is a bone derived protein mainly released by osteoblasts, and usually measured at the serum level as an indicator of bone formation (Hauschka et al., 1989). It is firstly produced as a pre-pro-peptide that become mature after the carboxylation of the three glutamic acid residues (Glu → Gla). Once OCN is mature and carboxylated it is released in the extracellular space to be incorporated into the bone ECM where it binds to hydroxyapatite (Poser et al., 1980; Malashkevich et al., 2013). In circulation there are several forms of OCN comprising the fully carboxylated (cOCN), the fully uncarboxylated (ucOCN) and the intermediate mono- and bi-carboxylated ones, together with several cleavage products, due to the susceptibility to enzymatic fragmentation, especially of the under-carboxylated forms (Lombardi et al., 2015).

Several studies in mice have shown a biological role for OCN, and mainly for ucOCN form, in metabolism. It has been shown that OCN regulates glucose metabolism, stimulates insulin release from islets, affects fertility and production of sex steroid hormones (Patti et al., 2013). Further, it has been reported to act on adipocytes where it stimulates the release of adiponectin (Kanazawa, 2015). Testis, skeletal muscles, liver, blood vessels, and small intestine have been identified as other targets of OCN activity. Actually, despite the evidence in rodents, and also in *in vitro* cell systems, such roles in humans have not been established and, also, current evidences indicate that the endocrine functions of OCN may be relegated to a mild, and possibly indirect, modulation (Lombardi et al., 2015).

During recent years, a new role emerged for OCN related to the nervous system, in rodents. Indeed, in mice OCN has been indicated as necessary for both brain development and function (Oury et al., 2013; Khrimian et al., 2017).

An *in vivo* mouse model of OCN−/− demonstrated a significant passivity, compared to the wild type (WT) counterpart, and the utility of OCN to correct brain development and cognition (Oury et al., 2013). Adult OCN−/− mice developed abnormalities in brain structures and alterations in neurotransmitter levels, impairment in learning and memory, and anxious-depressive phenotype. Further, after birth, mice with a complete deletion of OCN showed the same, or at least similar although less severe, phenotypic alterations, with the exception of brain morphological abnormalities, observed in adult OCN−/− mice. Intraventricular administration of OCN, in these mice, restored the normal phenotype and corrected anxiety and memory deficits (Oury et al., 2013). Experiments on this mouse model suggested that OCN may be necessary to reduce age-related cognitive impairment (Villeda et al., 2014). Interestingly, the circulating levels of OCN and cognitive functions strongly inversely correlate with age, suggesting that OCN may be necessary to contain the cognitive decline associated with aging.

Noteworthy, recent papers indicate that OCN can cross the BBB and, once in the CNS, it regulates neuronal functions, acting directly in brainstem, midbrain, and hippocampus to influence the synthesis of several neurotransmitters. It has been observed that, in OCN−/− mice, peripherally administrated ucOCN crosses the BBB and localizes at the brain level, while the cOCN form passes throughout the BBB less efficiently (Shan et al., 2019). Centrally, OCN stimulates the synthesis of monoamine neurotransmitters, including serotonin, dopamine, and noradrenaline, and inhibits the synthesis of γ-aminobutyric acid (GABA), that is the principal inhibitory neurotransmitter. Thus, OCN released from bone, cross the BBB and may have effects on the regulation of learning, memory, and cognitive functions (Oury et al., 2013).

Only recent clinical researches have been investigating on how peripheral level of OCN may put into correlation bone metabolism, and cognitive functions. It has been demonstrated a positive correlation between plasma OCN, executive functioning, and global cognition, but not with episode memory in women (Bradburn et al., 2016). Interestingly, no such correlation has been observed in men, suggesting the potential existence of gender differences in cognitive performance. Further, it has been reported that low levels of OCN correlate with brain microstructural changes observed with magnetic resonance imaging in middle-aged women (Puig et al., 2016).

#### Effects of Exercise

Circulating level of OCN increases after a single bout of endurance exercise in mice and, possibly, in humans (Mera et al., 2016), concomitantly to the increased glucose uptake by skeletal muscles and the reduction of circulating insulin concentrations. In particular, it has been observed that, in mice, this exercise-related increase of circulating OCN was associated with an increased bone resorption rate (Mera et al., 2016). Thus, aerobic exercise increases circulating levels of OCN, in mice, that may act through the IL-6/gpr130 axis to improve energy metabolism and cognition. In human, aerobic, and combined aerobic-resistance exercises increase serum level of OCN (Chahla et al., 2015; Mohammad Rahimi et al., 2020). These findings lead to the hypothesis that the induction of OCN release may associate with the preventive effect of exercise on dementia. However, additional studies are needed to better understand which form of OCN actually stimulates brain functions and to clarify which kind of exercise modulate a specific form of OCN. This would be of particular interest in the case of prescription of physical activity to specific cohorts of patients with the aim of ameliorating cognitive defects. At this purpose, it has been demonstrated that different kind of physical activity may associate with the relative prevalence of certain forms of OCN. During a 3-weeks stage race, professional cyclists, who underwent to strenuous activity in absence of loading, experienced a decrease in total circulating OCN without any change in the levels of ucOCN (i.e., decreased cOCN) in association with a stimulation of osteoclast activity (Lombardi et al., 2012b). On the contrary, male mountain ultra-marathoners, who are chronically subjected to strenuous activity associated with a high level of load, experienced circulating cOCN levels comparable to those recorded in their sedentary counterparts but halved ucOCN levels. Noteworthy, although a mountain ultra-marathon acutely induces a decrease in the circulating levels of bone formation markers, it causes a further decrease in ucOCN levels (Sansoni et al., 2017).

### Lipocalin2

Lipocalin2 is a glycoprotein that regulates several functions and, among them neutrophil response to pathogens (it is also known as neutrophil gelatinase-associated lipocalin), regulation of oxidative stress in kidney tubule cells, modulation of insulin release and energy metabolism. It has been recently discovered that, in normal conditions, at least 50% of circulating lipocalin2 (LCN2) is produced released by osteoblasts and this fraction is able to cross the BBB and to act in the hypothalamus. Indeed, throughout the generation of a mouse model lacking Lcn2 specifically in osteoblasts, Lcn2osb−/− (Mosialou et al., 2017), it has been demonstrated that osteoblasts are the cells that contribute LCN2 levels sufficient to regulate appetite and glucose metabolism, at least in basal states. This hypothesis has been further supported by the observation coming from several studies performed on mouse model in which the LCN2 was deleted from the germline cells. However, different studies have led to controversial results. Indeed, metabolic inflammation, has been closely associated with an increase of LCN2 suggesting a role of this hormone as pro-diabetic and pro-obesogenic. On the contrary, other studies evidenced an opposite role for LCN2, highlighting its functions as anti-diabetic and anti-obesogenic.

The increase of LCN2 during metabolic inflammation has been observed not only peripherally but also in the CNS and, particularly, in the hippocampus where it may solve a role in the modulation of inflammation (Bhusal et al., 2019). Interestingly, both peripheral and central administration of LCN2 reduce food intake and gain of body weight.

Lipocalin2 can cross the BBB and acts in hypothalamus where it activates neurons in the paraventricular nucleus. Further, series of molecular and biochemical studies showed that LCN2 activates the MC4R pathway, by directly binding to MC4R.

Regulation of appetite mediated by bone-derived LCN2 provides a feedback mechanism to the well-established central control of bone mass and therefore further illustrates how important is the cross-talk between bone and the brain.

#### Effects of Exercise

During bed rest, healthy volunteers experienced a time-dependent increase of circulating LCN2 levels. Further, LCN2 expression in bone was increased in mice subjected to mechanical unloading by tail suspension, or botulin toxin A-induced muscle paralysis, or in dystrophic mice, compared their normal loading/healthy counterparts. However, in these mice, exercise counteracted LCN2 increase (Rucci et al., 2015).

### Sclerostin

Sclerostin is a glycoprotein released by osteocytes that inhibits osteoblastogenesis through inhibition of Wnt-β catenin pathway and, hence sustains osteoclast-mediated bone resorption also throughout the concomitant induction of RANKL in osteoblasts (Li et al., 2005). Sclerostin is encoded by the *SOST* gene and its production is regulated by different factors, including mechanical stimulation. Indeed, it has been reported that mechanical loading decreases *SOST* transcription in osteocytes and, consequently, increases bone formation. Immobilization is associated with decreased BMD and increased serum sclerostin in animal models and humans (Robling et al., 2008; Spatz et al., 2013). However, the action of sclerostin within the skeleton is thought to reflect the local action of sclerostin released by osteocytes, while the amount of circulating sclerostin does not always reflect bone density. Indeed, the relationship between circulating sclerostin and bone formation is not simple and far from clear and, hence, further explorations of this aspect are required. Deficient sclerostin production in human causes sclerosteosis and Van Buchem disease both characterized by increase in bone mineral density (Brunkow et al., 2001; Balemans et al., 2002).

It has been demonstrated that sclerostin binds to the low-density lipoprotein-receptor-related protein-5 and -6 (LRP5/6) and inhibits the Wnt signaling (Li et al., 2005). Among the pleiotropic action of this pathway, Wnt signaling regulates synaptic plasticity and memory, and it has been linked to the pathogenesis neurodegenerative disorders, such as AD (Wan et al., 2014; Libro et al., 2016; Tapia-Rojas and Inestrosa, 2018). The hypothesis is that sclerostin could be involved in the regulation of this pathway in neurodegenerative diseases, however, it has not been yet demonstrated whether sclerostin is able to cross the BBB, or not. Other studies are necessary to understand if and how the modulation of the circulating levels of sclerostin (for instance, throughout specific exercise programs) can affect Wnt signaling in brain.

#### Effects of Exercise

Acute physical activity represents an important inhibitory stimulus for sclerostin (Lombardi et al., 2017). However, when this activity is performed in absence of load, especially when at high intensity, as in cycling, it may increase and associates to a state of stimulated bone resorption (Grasso et al., 2015). However, in professional athletes, who are chronically submitted to high-training workloads, at rest this stimulus is attenuated maybe due to negative feedback mechanism (Lombardi et al., 2012a) or dependently to the whole bone mass.

### Dickkopf-Related Protein 1

Dickkopf-related protein 1 protein is highly express in bone tissue, particularly it was first detected in osteoblasts and osteocytes, and it is secreted within the bone microenvironment (Li et al., 2006). Similarly to sclerostin, it has a role in bone mechanotransduction, and by binding to LRP6, it antagonizes Wnt/β-catenin pathway, thus inhibiting osteoblastogenesis, thus, favoring bone resorption (Li et al., 2006; Pinzone et al., 2009).

Notably, as mentioned above, Wnt signaling is fundamental for several functions, including regulation of synaptic plasticity, neuronal development, and functions, and its deregulation is associated with neurodegenerative disorders, such as AD.

Interestingly, Dkk1 protein has been found to be overexpressed in the brain of AD patients and transgenic-AD mice. In subsequent studies, investigating its specific role in neurodegeneration, demonstrated that the overexpression of Dkk1 protein in hippocampal mice causes impairment in long-term potentiation, learning and memory (Purro et al., 2012). Further, *in vivo* studies in rat and mouse and *in vitro* on neuronal cultures indicated that increased Dkk1 protein expression may contribute to cell death in cerebral ischemia, epilepsy, and neurodegenerative diseases (Cappuccio et al., 2005; Busceti et al., 2007; Mastroiacovo et al., 2009; Rosi et al., 2010). An interesting study measured peripheral Dkk1 levels in elderly women with memory concerns, in order to investigate a potential role as biomarker for Dkk1. This study revealed an inverse correlation between Dkk1 and cognitive performances but did not determine whether the serum level of Dkk1 corresponded to its brain level (Ross et al., 2018).

What is not clear is whether circulating Dkk1, released within bone microenvironment, can cross the BBB and can mediate this putative effect or, instead, a neural release of Dkk1 is responsible for the phenotypic findings.

#### Effects of Exercise

Dickkopf-related protein 1 serum level are decreased by long-term exercise (Kim et al., 2017), while during bed rest, exercise does not impede the unloading-associated increase in serum levels of sclerostin and Dkk1 (Belavy et al., 2016).

### Fibroblast Growth Factor 23

Fibroblast Growth Factor 23 is mostly expressed by bone osteoblasts and osteocytes even if its expression has been detected in small amounts also in some brain areas, such as hypothalamus, hippocampus, and cortex (Liu et al., 2006; Yoshiko et al., 2007) and it has also been detected in the cerebrospinal fluid (CSF) (Kunert et al., 2017). However, the majority of circulating fibroblast growth factor 23 (FGF23) derives from osteocytes (Feng et al., 2006). When unstimulated (i.e., unloaded) osteocytes expresses FGF23, together with sclerostin and Dkk1. This response on one hand, inhibits osteoblast synthetic activity and differentiation, thus, favoring osteoclast-mediated bone resorption, on the other side make the other organs aware about the increased circulating load of bone catabolic products (e.g., calcium and phosphorous). Indeed, FGF23 acts on the kidney tubule where it stimulates the excretion of phosphorous in urine and also regulates vitamin D metabolism (Lombardi et al., 2014).

Despite the reported expression and detection in brain, definitive roles for FGF23 in brain functioning have not yet been established.

*In vitro*, murine hippocampal neurons treated with medium enriched with FGF23 show a less complex morphology (Hensel et al., 2016). A recent paper using a FGF23 knockout mouse model has demonstrated an impairment of hippocampal-dependent cognitive functions without any structural brain alterations (Laszczyk et al., 2019).

Interestingly, there are various papers that demonstrate that all the compounds belonging to the FGF family can cross the BBB and, consequently, it has been hypothesized a similar feature for FGF23 (Cuevas et al., 1998; Hsuchou et al., 2013).

#### Effects of Exercise

Serum levels of FGF23 decrease in mice subjected to both acute and chronic physical exercises and this is in accordance with FGF23 role on skeletal mineralization (Li et al., 2016). During a 3-weeks stage race in cyclists, who experience strenuous skeletal muscle activity in absence of load, FGF23 serum levels increased in association to enhanced osteoclast activity and parallel rise in serum phosphorous level (Lombardi et al., 2014). However, another study performed on rats demonstrated no changes in FGF23 serum levels after peak power and endurance training, demonstrating that further studies are necessary to better investigate on FGF23 serum level modulation after physical activity (Buskermolen et al., 2019).

## Other Mediators in the Brain-Bone-Brain Cross-Talk

There are several key molecules that are expressed and exert functions on both brain and bone. Thus, in the absence of specific studies, it is difficult to understand if these molecules act only locally or if bone derived molecules could cross the BBB and act also in brain or *vice versa*. Among these molecules one can account irisin (whose roles have been discussed above), OPN, RANKL, bone morphogenic protein (BMP), brain-derived neurotrophic factor (BDNF), and IGF-1.

### Osteopontin

Osteopontin is a bone-derived glycoprotein that is expressed also by other different tissues and organs and is a known mediator of the osteo-immune crosstalk (Lanteri et al., 2012). Evidences indicate that OPN is also expressed in the brain. In bone it is present in the ECM and promotes bone resorption supporting bone demineralization by anchoring osteoclasts to bone mineral matrix (Reinholt et al., 1990; Singh et al., 2018). It has been observed that patients with high serum level of OPN have low BMD (Filardi et al., 2019).

In brain it seems to protect neurons and regulate repair processes in various brain disorders and neurodegenerative diseases (Kaleta, 2019). Interestingly, OPN protein levels were found high in patient with AD in both CSF and plasma, and more elevated in newly diagnosed AD compared to chronic patients (Comi et al., 2010; Sun et al., 2013). It has also been found highly expressed in brain sections of subjects affected by PD (Maetzler et al., 2007).

### Receptor Activator of Nuclear Factor κB

Receptor activator of nuclear factor κB ligand, as mentioned above, binds RANK expressed on osteoclasts and osteoclasts precursors and activates their differentiation into mature resorbing cells (Liu and Zhang, 2015). It is expressed in various tissues including bone and brain.

In bone, it favors bone resorption process, and it is an established therapeutic target for in the treatment of osteoporosis. Indeed, anti-RANKL antibody, that blocks RANKL–RANK binding, is currently successfully used as an anti-resorptive treatment.

In brain, RANKL is highly express in the hypothalamus and its function is to control the central regulation of body temperature and fever, in females (Hanada et al., 2009). Interestingly, the treatment with anti-RANKL in mice affected by chronic social defeat stress and depression-like syndrome ameliorates the phenotypes, suggesting a putative novel therapeutic use of anti-RANKL antibodies in human depression (Zhang et al., 2020).

### Bone Morphogenic Proteins

Bone morphogenic proteins (BMPs) are growth factors belonging to the largest transforming growth factor β (TGFβ) superfamily and are expressed by different tissues, including bone and brain. These proteins are extracellular multifunctional cytokines that could be sequestered in the ECM during its deposition. Indeed, in ECM there is a high concentration of BMP ligands and also of antagonists of BMPs that inhibit their activity through transmembrane serine/threonine kinase receptors. Hence, the action of BMPs entrapped in ECM is finely regulated by agonists or antagonists (Weiss and Attisano, 2013).

In bone, BMPs stimulate skeletal growth, promoting bone formation and remodeling. BMPs signaling is modulated by different mechanostimuli leading to a specific BMP action. Since sequestered in ECM, BMPs are in a dynamically active space responding to different mechanical stimuli. For instance, fluid shear stress determines an immediate increase of BMPs signaling, consequent to their release from ECM, that stimulates osteocytes and osteoblasts (Schreivogel et al., 2019). Thus, mechanically modulated BMPs affect osteogenesis. However, it is important to keep in mind that several other molecules exist that are intermediates between mechanosensing and BMPs signaling (da Silva Madaleno et al., 2020). The mechanosensitive growth factor MGF24E is one of these molecules; it stimulates SMAD phosphorylation and the expression of osteogenic genes, finally increasing bone mineral density (Deng et al., 2015). This consideration is important in view of therapeutic approaches whose aim is to stimulate bone regeneration through the modulation of BMPs levels.

It is known that in adult brain, exists a niche that hosts neural stem cells the subgranular zone of the hippocampal dentate gyrus, where adult neurogenesis persists. As described above adult neurogenesis could be stimulated by physical activity, since it stimulates bone molecules that consequently could act in brain. BMPs in brain are involved in neurogenesis both in embryonic stages and in adulthood, by acting at that level (Gobeske et al., 2009; Armenteros et al., 2018; Jovanovic et al., 2018). Thus, these molecules could be considered as novel therapeutic targets for those pathologies in which bone loss associates to the neurodegenerative disorder, since they may stimulate both neurogenesis and bone anabolism.

### Brain-Derived Neurotrophic Factor

Brain-derived neurotrophic factor is a neurotrophic factor that is released by CNS and PNS. Further, it could be expressed and released by other tissues, such as bone. In brain, it solves several roles, including support to synaptic plasticity, neurodevelopment, and neuronal differentiation. It has been observed that its levels are reduced in patients affected by neurodegenerative diseases, such as AD and PD (Mattson, 2008).

As mentioned above, BDNF and its receptors are also expressed in osteoblasts and chondrocyte. *In vitro* experiments showed the beneficial effects of BDNF on bone cells promoting differentiation of MSC into osteoblasts (Kauschke et al., 2018), while conditional knockout mice for BDNF in brain displayed defects in bone, including high bone mass and longer femurs (Camerino et al., 2012).

### Insulin-Like Growth Factor 1

Insulin-Like Growth Factor 1 (IGF-1) is a hormone structurally similar to insulin, which is primarily synthetized in liver following GH signaling (Laviola et al., 2008). Besides liver, it is synthesized in other tissues, including bone and brain. IGF-1 signaling is central to pathways that promote cell growth and survival, maturation, and proliferation, allowing for tissue growth and renewal. Indeed it is highly express in all neuroephitelial cell type during embryogenesis and in adulthood in those brain areas where persist neurogenesis (cerebellum, olfactory bulb, and hippocampus) (Bach et al., 1991; Bartlett et al., 1991; Bondy et al., 1992).

In bone it stimulates chondrocyte proliferation and osteoblast differentiation, thus promoting bone formation. Indeed, it has been found that IGF-1 serum levels are decreased with age and in patients with osteoporosis (Yakar et al., 2002; Rosen, 2004; Wrigley et al., 2017).

In brain IGF-1 has roles in neurodevelopment both prenatally and in the early post-natal period, and in plasticity and remodeling throughout the life. Noteworthy, this hormone is involved in neuropsychiatric and neurodegenerative disorders associated with aging (Cohen et al., 2009; Bozdagi et al., 2013; Shcheglovitov et al., 2013; Gontier et al., 2015).

## Conclusion

### Brain Diseases: Biomechanical Intervention as a Support to the Therapy

Different studies have demonstrated that there is a strong correlation between cognitive impairment and bone diseases. Impaired cognitive functions and neurodegeneration, indeed, are often associated with defects in bone (Roos, 2014; Binks and Dobson, 2016), while therapeutic strategies addressed at improving bone status (e.g., exercise training) associate with lower risk of cognitive impairment or dementia (Lee et al., 2019).

Brain and neurodegenerative diseases, independently from the involvement of skeletal muscles, associate with a limited physical activity behavior that results in an impaired bone function (i.e., osteopenia and osteoporosis). However, exercise would have a beneficial effect on bone function, and beside the positive impact on brain function dependent upon the improvement in energy metabolism, bone (and skeletal muscle)-derived mediators may act on, and positively affect, central PNSs. These bone-derived mediators can be induced by specifically addressed exercise training. Thereby, taking advantage of the existing connection between bone and brain, a novel view is emerging about the possibility to improve brain functions throughout the stimulation of bone metabolism.

It has been shown that low bone density (BMD) and osteoporosis may associate with dementia and AD in postmenopausal women (Filardi et al., 2019; Kaleta, 2019). Further, low BMD and BMD loss are risk factors for osteoporosis and AD and is an early risk factor for dementia. Women with high levels of hip bone loss have an increased probability to develop cognitive dementia compared to women with limited loss. In addition, looking at this point from another point of view, it has been shown that high lean body mass is associated with lower risk of cognitive impairment or dementia (Maetzler et al., 2007; Comi et al., 2010; Sun et al., 2013).

It is well known that physical activity decreases the risk for several diseases and improves the quality of life. Several studies have shown that physical exercise could prevent or even ameliorate cognitive impairment and dementia conditions, but also can bring benefits to memory and brain functions (Hillman et al., 2008; Heisz et al., 2015). Besides exercise, also healthy lifestyle, such as good eating habits, and adequate sleep time, prevent dementia. The prevention of dementia throughout physical activity has been demonstrated by a study that showed that exercise could promote the transcription of those genes that regulate the production of free radical scavenging enzymes and preventing free radical-mediated damage to neurons (Simioni et al., 2018; De la Rosa et al., 2020). Further, it has been observed that exercising ameliorates the cognitive functions by improving hippocampal volume and increasing mitochondria biogenesis in neurons, thus, favoring the energy metabolism in neurons (Olson et al., 2006; Fabel et al., 2009; Steiner et al., 2011).

It has been recently proposed that the improvement in brain functions consequent to physical exercise could be associated with the activity of molecules released by bone in response to the biomechanical stimulation. And, indeed, it is known that huge amounts of mediators are released in the bloodstream after exercises and most of them have specific positive effects on neurogenesis, angiogenesis, synaptic plasticity, and hippocampal dendritic spine densities, as well as in maintaining and improving the cognitive function (Katsimpardi et al., 2014; Heisz et al., 2017).

As describe in detail above, the circulating levels of bone-derived molecules, i.e., osteokines, change depending on the loading status and condition. LCN2 peripheral levels are inversely associated with the exercising status: less exercise corresponds to high level of LCN2 and, consequently, to reduce the brain-determined caloric intake. OCN circulating levels are also modulated by exercises highlighting its potentially therapeutic modulation for improving age-related cognitive decline, preventing anxiety and depression, and preventing neurodegeneration in those pathologies affected by neuronal loss, as PD and AD.

Regulation of sclerostin and Dkk1 circulating levels by exercise is important for regulation of bone remodeling. Circulating sclerostin levels seem to inversely correlate with physical exercise, decreasing after mechanical stimuli to inhibit bone resorption. It emerges an important role of Wnt signaling in bone remodeling and this pathway is highly modulated by loading and unloading (Robling and Turner, 2009; Duan and Bonewald, 2016; Galea et al., 2017). Thus, pharmacotherapy targeting Wnt/β-catenin signaling may be useful in combination with load bearing exercise programs.

IGF-1 and BDNF are two neurotrophins that potentially could be used as biomarkers for recovery of patient suffering of ischemic stroke or other neurodegenerative pathologies, but the precise molecular mechanisms by which their levels can be regulated by physical activity are not definitively understood (Carro et al., 2000; Trejo et al., 2001; Huang et al., 2014; Maass et al., 2016).

Importantly, physical exercise improves cognitive functions by producing positive benefits whose impact may be further enhanced throughout the combination with a specific cognitive training. Indeed, synergistic effects of physical and cognitive exercises on memory function and serum levels of neurotrophic molecules, particularly of IGF-1 and BDNF, have been described in young adults and their levels were associated with positive results in those subjects experiencing the better responses to exercises (Heisz et al., 2017).

### Future Perspectives

With this review we have highlighted the emerging aspects of the complex interconnection that exists between bone and brain. From this overview, it emerges a dynamic role of bone as a mechanosensor and endocrine organ, able to respond to mechanostimulation throughout the release of molecules whose function is to coordinate a proper adoptive response to the changing environmental situations. Physical exercise, indeed, perturbs bone homeostasis and stimulates bone response by determining benefit for the bone itself and, directly or indirectly, for the brain. However, if, from one hand, the knowledge of the biological role of some mediators in the context of the brain-to-brain crosstalk are somehow advanced, for others mediators such a function has been just hypothesized and, however, far to be demonstrated. As a matter of fact, the presentation of these factor results imbalanced.

As described above, patients affected by both brain diseases very often experience also bone metabolic dysfunctions. Exploiting the influence of bone on brain, novel therapeutic interventions, possibly exercise-based adjuvant strategies to the standard pharmacological and psychiatric treatments, may be adopted to treat neurological pathologies. Such strategies should be addressed at modulating the circulating level of specific bone-derived molecules, throughout specific exercise programs, in order to obtain positive effects also at the central level. Such an approach, however, in order to be effective needs a further and deep investigation of the mechanisms underlying the bone-to-brain axis and the future researches should answer to the following questions: which kind of stimulus can specifically modulate the expression of a given mediator?; is this mediator able to cross the BBB? and in which measure?; how does this mediator act in brain?

## Author Contributions

LG and GL drafted the manuscript and critically revised the manuscript. Both authors contributed to the article and approved the submitted version.

## Conflict of Interest

The authors declare that the research was conducted in the absence of any commercial or financial relationships that could be construed as a potential conflict of interest.

## References

[B1] Abe E. Marians R. C. Yu W. Wu X. B. Ando T. Li Y. (2003). TSH is a negative regulator of skeletal remodeling. *Cell* 115 151–162. 10.1016/s0092-8674(03)00771-2 14567913

[B2] Akoume M. Y. Elbakry M. Veillette M. Franco A. Nada D. Labelle H. (2019). A Differential Hypofunctionality of Galphai Proteins Occurs in Adolescent Idiopathic Scoliosis and Correlates with the Risk of Disease Progression. *Sci. Rep.* 9:10074. 10.1038/s41598-019-46325-2 31296888 PMC6624302

[B3] Anastasilakis A. D. Polyzos S. A. Makras P. Gkiomisi A. Bisbinas I. Katsarou A. (2014). Circulating irisin is associated with osteoporotic fractures in postmenopausal women with low bone mass but is not affected by either teriparatide or denosumab treatment for 3 months. *Osteoporos Int.* 25 1633–1642. 10.1007/s00198-014-2673-x 24599275

[B4] Armenteros T. Andreu Z. Hortiguela R. Lie D. C. Mira H. (2018). BMP and WNT signalling cooperate through LEF1 in the neuronal specification of adult hippocampal neural stem and progenitor cells. *Sci. Rep.* 8:9241. 10.1038/s41598-018-27581-0 29915186 PMC6006330

[B5] Asadi Y. Gorjipour F. Behrouzifar S. Vakili A. (2018). Irisin Peptide Protects Brain Against Ischemic Injury Through Reducing Apoptosis and Enhancing BDNF in a Rodent Model of Stroke. *Neurochem. Res.* 43 1549–1560. 10.1007/s11064-018-2569-9 29882126

[B6] Bach M. A. Shen-Orr Z. Lowe W. L. Jr. Roberts C. T. Jr. LeRoith D. (1991). Insulin-like growth factor I mRNA levels are developmentally regulated in specific regions of the rat brain. *BrainRes. Mol. Brain Res.* 10 43–48. 10.1016/0169-328x(91)90054-2 1647481

[B7] Bajayo A. Bar A. Denes A. Bachar M. Kram V. Attar-Namdar M. (2012). Skeletal parasympathetic innervation communicates central IL-1 signals regulating bone mass accrual. *Proc. Nat. Acad. Sci. USA* 109 15455–15460. 10.1073/pnas.1206061109 22949675 PMC3458367

[B8] Baldock P. A. Lee N. J. Driessler F. Lin S. Allison S. Stehrer B. (2009). Neuropeptide Y knockout mice reveal a central role of NPY in the coordination of bone mass to body weight. *PLoS One* 4:e8415. 10.1371/journal.pone.0008415 20027231 PMC2794533

[B9] Balemans W. Patel N. Ebeling M. Van Hul E. Wuyts W. Lacza C. (2002). Identification of a 52 kb deletion downstream of the SOST gene in patients with van Buchem disease. *J. Med. Genet.* 39 91–97. 10.1136/jmg.39.2.91 11836356 PMC1735035

[B10] Baliram R. Chow A. Huber A. K. Collier L. Ali M. R. Morshed S. A. (2013). Thyroid and bone: macrophage-derived TSH-beta splice variant increases murine osteoblastogenesis. *Endocrinology* 154 4919–4926. 10.1210/en.2012-2234 24140716 PMC3836071

[B11] Baliram R. Latif R. Berkowitz J. Frid S. Colaianni G. Sun L. (2011). Thyroid-stimulating hormone induces a Wnt-dependent, feed-forward loop for osteoblastogenesis in embryonic stem cell cultures. *Proc. Nat. Acad. Sci. USA* 108 16277–16282. 10.1073/pnas.1110286108 21911383 PMC3182731

[B12] Barr V. A. Malide D. Zarnowski M. J. Taylor S. I. Cushman S. W. (1997). Insulin stimulates both leptin secretion and production by rat white adipose tissue. *Endocrinology* 138 4463–4472. 10.1210/endo.138.10.5451 9322964

[B13] Bartlett W. P. Li X. S. Williams M. Benkovic S. (1991). Localization of insulin-like growth factor-1 mRNA in murine central nervous system during postnatal development. *Dev. Biol.* 147 239–250. 10.1016/s0012-1606(05)80021-1 1879610

[B14] Basgoz B. Ince S. Safer U. Naharci M. I. Tasci I. (2020). Low bone density and osteoporosis among older adults with Alzheimer’s disease, vascular dementia, and mixed dementia: A Cross-sectional Study With Prospective Enrollment. *Turk J. Phs. Med. Rheabil.* 66 193–200. 10.5606/tftrd.2020.3803 32760897 PMC7401688

[B15] Belavy D. L. Baecker N. Armbrecht G. Beller G. Buehlmeier J. Frings-Meuthen P. (2016). Serum sclerostin and DKK1 in relation to exercise against bone loss in experimental bed rest. *J. Bone Miner Metab.* 34 354–365. 10.1007/s00774-015-0681-3 26056021

[B16] Bergwitz C. Juppner H. (2010). Regulation of phosphate homeostasis by PTH, vitamin D, and FGF23. *Ann. Rev. Med.* 61 91–104. 10.1146/annurev.med.051308.111339 20059333 PMC4777331

[B17] Bhangu P. S. (2003). ‘Pre-synaptic’ vesicular glutamate release mechanisms in osteoblasts. *J. Musculoskelet. Neuronal. Interact.* 3 17–29. 15758362

[B18] Bhusal A. Rahman M. H. Lee W. H. Bae Y. C. Lee I. K. Suk K. (2019). Paradoxical role of lipocalin-2 in metabolic disorders and neurological complications. *Biochem. Pharmacol.* 169:113626. 10.1016/j.bcp.2019.113626 31476294

[B19] Binks S. Dobson R. (2016). Risk Factors, Epidemiology and Treatment Strategies for Metabolic Bone Disease in Patients with Neurological Disease. *Curr. Osteoporos. Rep.* 14 199–210. 10.1007/s11914-016-0320-5 27525980

[B20] Bliziotes M. Eshleman A. Burt-Pichat B. Zhang X. W. Hashimoto J. Wiren K. (2006). Serotonin transporter and receptor expression in osteocytic MLO-Y4 cells. *Bone* 39 1313–1321. 10.1016/j.bone.2006.06.009 16884969 PMC1766480

[B21] Bondy C. Werner H. Roberts C. T. Jr. LeRoith D. (1992). Cellular pattern of type-I insulin-like growth factor receptor gene expression during maturation of the rat brain: comparison with insulin-like growth factors I and II. *Neurosci.* 46 909–923. 10.1016/0306-4522(92)90193-6 1311816

[B22] Bonewald L. F. (2017). The Role of the Osteocyte in Bone and Nonbone Disease. *Endocrinol. Metab. Clin. North Am.* 46 1–18. 10.1016/j.ecl.2016.09.003 28131126 PMC5300041

[B23] Bostrom P. Wu J. Jedrychowski M. P. Korde A. Ye L. Lo J. C. (2012). A PGC1-alpha-dependent myokine that drives brown-fat-like development of white fat and thermogenesis. *Nature* 481 463–468. 10.1038/nature10777 22237023 PMC3522098

[B24] Bozdagi O. Tavassoli T. Buxbaum J. D. (2013). Insulin-like growth factor-1 rescues synaptic and motor deficits in a mouse model of autism and developmental delay. *Mol. Autism.* 4:9. 10.1186/2040-2392-4-9 23621888 PMC3649942

[B25] Bradburn S. McPhee J. S. Bagley L. Sipila S. Stenroth L. Narici M. V. (2016). Association between osteocalcin and cognitive performance in healthy older adults. *Age Ageing* 45 844–849. 10.1093/ageing/afw137 27515675 PMC5105824

[B26] Brazill J. M. Beeve A. T. Craft C. S. Ivanusic J. J. Scheller E. L. (2019). Nerves in Bone: Evolving Concepts in Pain and Anabolism. *J. Bone Miner Res.* 34 1393–1406. 10.1002/jbmr.3822 31247122 PMC6697229

[B27] Brunkow M. E. Gardner J. C. Van Ness J. Paeper B. W. Kovacevich B. R. Proll S. (2001). Bone dysplasia sclerosteosis results from loss of the SOST gene product, a novel cystine knot-containing protein. *Am. J. Hum. Genet.* 68 577–589. 10.1086/318811 11179006 PMC1274471

[B28] Burns J. M. Johnson D. K. Watts A. Swerdlow R. H. Brooks W. M. (2010). Reduced lean mass in early Alzheimer disease and its association with brain atrophy. *Archiv. Neurol.* 67 428–433. 10.1001/archneurol.2010.38 20385908 PMC2855150

[B29] Busceti C. L. Biagioni F. Aronica E. Riozzi B. Storto M. Battaglia G. (2007). Induction of the Wnt inhibitor, Dickkopf-1, is associated with neurodegeneration related to temporal lobe epilepsy. *Epilepsia* 48 694–705. 10.1111/j.1528-1167.2007.01055.x 17437412

[B30] Buskermolen J. van der Meijden K. Furrer R. Mons D. J. van Essen H. W. Heijboer A. C. (2019). Effects of different training modalities on phosphate homeostasis and local vitamin D metabolism in rat bone. *PeerJ.* 7:e6184. 10.7717/peerj.6184 30697476 PMC6348094

[B31] Calvi L. M. Adams G. B. Weibrecht K. W. Weber J. M. Olson D. P. Knight M. C. (2003). Osteoblastic cells regulate the haematopoietic stem cell niche. *Nature* 425 841–846. 10.1038/nature02040 14574413

[B32] Camerino C. Zayzafoon M. Rymaszewski M. Heiny J. Rios M. Hauschka P. V. (2012). Central depletion of brain-derived neurotrophic factor in mice results in high bone mass and metabolic phenotype. *Endocrinology* 153 5394–5405. 10.1210/en.2012-1378 23011922 PMC3685798

[B33] Cappariello A. Ponzetti M. Rucci N. (2016). The “soft” side of the bone: unveiling its endocrine functions. *Horm. Mol. Biol. Clin. Invest.* 28 5–20. 10.1515/hmbci-2016-0009 27107839

[B34] Cappuccio I. Calderone A. Busceti C. L. Biagioni F. Pontarelli F. Bruno V. (2005). Induction of Dickkopf-1, a negative modulator of the Wnt pathway, is required for the development of ischemic neuronal death. *J. Neurosci.* 25 2647–2657. 10.1523/JNEUROSCI.5230-04.2005 15758175 PMC6725177

[B35] Cardoso L. Herman B. C. Verborgt O. Laudier D. Majeska R. J. Schaffler M. B. (2009). Osteocyte apoptosis controls activation of intracortical resorption in response to bone fatigue. *J. Bone Miner. Res.* 24 597–605. 10.1359/jbmr.081210 19049324 PMC2659511

[B36] Carro E. Nunez A. Busiguina S. Torres-Aleman I. (2000). Circulating insulin-like growth factor I mediates effects of exercise on the brain. *J. Neurosci.* 20 2926–2933. 10751445 10.1523/JNEUROSCI.20-08-02926.2000 PMC6772191

[B37] Chahla S. E. Frohnert B. I. Thomas W. Kelly A. S. Nathan B. M. Polgreen L. E. (2015). Higher daily physical activity is associated with higher osteocalcin levels in adolescents. *Prev. Med. Rep.* 2 568–571. 10.1016/j.pmedr.2015.06.017 26236583 PMC4517293

[B38] Chen C. S. Alonso J. L. Ostuni E. Whitesides G. M. Ingber D. E. (2003). Cell shape provides global control of focal adhesion assembly. *Biochem. Biophys. Res. Commun.* 307 355–361. 10.1016/s0006-291x(03)01165-3 12859964

[B39] Chen C. Y. Lane H. Y. Lin C. H. (2016). Effects of Antipsychotics on Bone Mineral Density in Patients with Schizophrenia: Gender Differences. *Clin. Psychopharmacol. Neurosci.* 14 238–249. 10.9758/cpn.2016.14.3.238 27489377 PMC4977815

[B40] Chen H. Senda T. Kubo K. Y. (2015). The osteocyte plays multiple roles in bone remodeling and mineral homeostasis. *Med. Mol. Morphol.* 48 61–68. 10.1007/s00795-015-0099-y 25791218

[B41] Chenu C. Serre C. M. Raynal C. Burt-Pichat B. Delmas P. D. (1998). Glutamate receptors are expressed by bone cells and are involved in bone resorption. *Bone* 22 295–299. 10.1016/s8756-3282(97)00295-0 9556127

[B42] Cohen E. Paulsson J. F. Blinder P. Burstyn-Cohen T. Du D. Estepa G. (2009). Reduced IGF-1 signaling delays age-associated proteotoxicity in mice. *Cell* 139 1157–1169. 10.1016/j.cell.2009.11.014 20005808 PMC3017511

[B43] Colaianni G. Cuscito C. Mongelli T. Pignataro P. Buccoliero C. Liu P. (2015). The myokine irisin increases cortical bone mass. *Proc. Nat. Acad. Sci. USA* 112 12157–12162. 10.1073/pnas.1516622112 26374841 PMC4593131

[B44] Comi C. Carecchio M. Chiocchetti A. Nicola S. Galimberti D. Fenoglio C. (2010). Osteopontin is increased in the cerebrospinal fluid of patients with Alzheimer’s disease and its levels correlate with cognitive decline. *J. Alzheimer. Dis.* 19 1143–1148. 10.3233/JAD-2010-1309 20308780

[B45] Cuevas P. Carceller F. Munoz-Willery I. Gimenez-Gallego G. (1998). Intravenous fibroblast growth factor penetrates the blood-brain barrier and protects hippocampal neurons against ischemia-reperfusion injury. *Surg. Neurol.* 49 77–83. 10.1016/s0090-3019(97)00193-6 9428898

[B46] Cui Y. Niziolek P. J. MacDonald B. T. Zylstra C. R. Alenina N. Robinson D. R. (2011). Lrp5 functions in bone to regulate bone mass. *Nat. Med.* 17 684–691. 10.1038/nm.2388 21602802 PMC3113461

[B47] da Silva Madaleno C. Jatzlau J. Knaus P. (2020). BMP signalling in a mechanical context - Implications for bone biology. *Bone* 137:115416. 10.1016/j.bone.2020.115416 32422297

[B48] Dallas S. L. Prideaux M. Bonewald L. F. (2013). The osteocyte: an endocrine cell. and more. *Endocrine Rev.* 34 658–690. 10.1210/er.2012-1026 23612223 PMC3785641

[B49] David V. Martin A. Lafage-Proust M. H. Malaval L. Peyroche S. Jones D. B. (2007). Mechanical loading down-regulates peroxisome proliferator-activated receptor gamma in bone marrow stromal cells and favors osteoblastogenesis at the expense of adipogenesis. *Endocrinology* 148 2553–2562. 10.1210/en.2006-1704 17317771

[B50] De la Rosa A. Olaso-Gonzalez G. Arc-Chagnaud C. Millan F. Salvador-Pascual A. Garcia-Lucerga C. (2020). Physical exercise in the prevention and treatment of Alzheimer’s disease. *J. Sport Health Sci.* 9 394–404. 10.1016/j.jshs.2020.01.004 32780691 PMC7498620

[B51] Denes A. Boldogkoi Z. Uhereczky G. Hornyak A. Rusvai M. Palkovits M. (2005). Central autonomic control of the bone marrow: multisynaptic tract tracing by recombinant pseudorabies virus. *Neurosci* 134 947–963. 10.1016/j.neuroscience.2005.03.060 15994021

[B52] Deng M. Liu P. Xiao H. Zhang Y. Wang Y. Zhao J. (2015). Improving the osteogenic efficacy of BMP2 with mechano growth factor by regulating the signaling events in BMP pathway. *Cell Tissue Res.* 361 723–731. 10.1007/s00441-015-2154-3 25843688

[B53] DiGirolamo D. J. Mukherjee A. Fulzele K. Gan Y. Cao X. Frank S. J. (2007). Mode of growth hormone action in osteoblasts. *J. Biol. Chem.* 282 31666–31674. 10.1074/jbc.M705219200 17698843

[B54] Ding Y. Arai M. Kondo H. Togari A. (2010). Effects of capsaicin-induced sensory denervation on bone metabolism in adult rats. *Bone* 46 1591–1596. 10.1016/j.bone.2010.02.022 20193788

[B55] Dobie R. MacRae V. E. Huesa C. van’t Hof R. Ahmed S. F. Farquharson C. (2014). Direct stimulation of bone mass by increased GH signalling in the osteoblasts of Socs2-/- mice. *J. Endocrinol.* 223 93–106. 10.1530/JOE-14-0292 25074853 PMC4166176

[B56] Duan P. Bonewald L. F. (2016). The role of the wnt/beta-catenin signaling pathway in formation and maintenance of bone and teeth. *Int. J. Cel. Biochem. Cell Biol.* 77 23–29. 10.1016/j.biocel.2016.05.015 27210503 PMC4958569

[B57] Dumont L. M. Wu C. S. Tatnell M. A. Cornish J. Mountjoy K. G. (2005). Evidence for direct actions of melanocortin peptides on bone metabolism. *Peptides* 26 1929–1935. 10.1016/j.peptides.2004.12.034 15979763

[B58] Duncan R. L. Turner C. H. (1995). Mechanotransduction and the functional response of bone to mechanical strain. *Calcif Tissue Int.* 57 344–358. 10.1007/BF00302070 8564797

[B59] Dupont S. Morsut L. Aragona M. Enzo E. Giulitti S. Cordenonsi M. (2011). Role of YAP/TAZ in mechanotransduction. *Nature* 474 179–183. 10.1038/nature10137 21654799

[B60] Elefteriou F. (2005). Neuronal signaling and the regulation of bone remodeling. *Cell Mol. Life Sci.* 62 2339–2349. 10.1007/s00018-005-5175-3 16132233 PMC11139174

[B61] Elefteriou F. (2008). Regulation of bone remodeling by the central and peripheral nervous system. *Archiv. Biochem. Biophys.* 473 231–236. 10.1016/j.abb.2008.03.016 18410742 PMC2430105

[B62] Elefteriou F. Ahn J. D. Takeda S. Starbuck M. Yang X. Liu X. (2005). Leptin regulation of bone resorption by the sympathetic nervous system and CART. *Nature* 434 514–520. 10.1038/nature03398 15724149

[B63] En-Nosse M. Hartmann S. Trinkaus K. Alt V. Stigler B. Heiss C. (2009). Expression of non-neuronal cholinergic system in osteoblast-like cells and its involvement in osteogenesis. *Cell Tissue Res.* 338 203–215. 10.1007/s00441-009-0871-1 19820967

[B64] Fabel K. Wolf S. A. Ehninger D. Babu H. Leal-Galicia P. Kempermann G. (2009). Additive effects of physical exercise and environmental enrichment on adult hippocampal neurogenesis in mice. *Front. Neurosci.* 3:50. 10.3389/neuro.22.002.2009 20582277 PMC2858601

[B65] Faraldi M. Gomarasca M. Perego S. Sansoni V. Banfi G. Lombardi G. (2020). Effect of collection matrix, platelet depletion, and storage conditions on plasma extracellular vesicles and extracellular vesicle-associated miRNAs measurements. *Clin. Chem. Lab. Med.* 2020:1296. 10.1515/cclm-2020-1296 33555147

[B66] Farooqi I. S. Yeo G. S. Keogh J. M. Aminian S. Jebb S. A. Butler G. (2000). Dominant and recessive inheritance of morbid obesity associated with melanocortin 4 receptor deficiency. *J. Clin. Invest.* 106 271–279. 10.1172/JCI9397 10903343 PMC314308

[B67] Feng J. Q. Ward L. M. Liu S. Lu Y. Xie Y. Yuan B. (2006). Loss of DMP1 causes rickets and osteomalacia and identifies a role for osteocytes in mineral metabolism. *Nat. Genet.* 38 1310–1315. 10.1038/ng1905 17033621 PMC1839871

[B68] Filardi T. Carnevale V. Massoud R. Russo C. Nieddu L. Tavaglione F. (2019). High serum osteopontin levels are associated with prevalent fractures and worse lipid profile in post-menopausal women with type 2 diabetes. *J. Endocrinol. Invest.* 42 295–301. 10.1007/s40618-018-0914-0 29916137

[B69] Florencio-Silva R. Sasso G. R. Sasso-Cerri E. Simoes M. J. Cerri P. S. (2015). Biology of Bone Tissue: Structure, Function, and Factors That Influence Bone Cells. *BioMed. Res. Int.* 2015 421746. 10.1155/2015/421746 26247020 PMC4515490

[B70] Forouzanfar M. Rabiee F. Ghaedi K. Beheshti S. Tanhaei S. Shoaraye Nejati A. (2015). Fndc5 overexpression facilitated neural differentiation of mouse embryonic stem cells. *Cell Biol. Int.* 39 629–637. 10.1002/cbin.10427 25572300

[B71] Friedman S. M. Menzies I. B. Bukata S. V. Mendelson D. A. Kates S. L. (2010). Dementia and hip fractures: development of a pathogenic framework for understanding and studying risk. *Geriatr. Orthop. Surg. Rehabil.* 1 52–62. 10.1177/2151458510389463 23569663 PMC3597300

[B72] Fukuda T. Takeda S. Xu R. Ochi H. Sunamura S. Sato T. (2013). Sema3A regulates bone-mass accrual through sensory innervations. *Nature* 497 490–493. 10.1038/nature12115 23644455

[B73] Galea G. L. Lanyon L. E. Price J. S. (2017). Sclerostin’s role in bone’s adaptive response to mechanical loading. *Bone* 96 38–44. 10.1016/j.bone.2016.10.008 27742499 PMC5340132

[B74] Galli C. Piemontese M. Lumetti S. Manfredi E. Macaluso G. M. Passeri G. (2012). The importance of WNT pathways for bone metabolism and their regulation by implant topography. *Eur. Cell Mater* 24 46–59. 10.22203/ecm.v024a04 22791372

[B75] Gobeske K. T. Das S. Bonaguidi M. A. Weiss C. Radulovic J. Disterhoft J. F. (2009). BMP signaling mediates effects of exercise on hippocampal neurogenesis and cognition in mice. *PLoS One* 4:e7506. 10.1371/journal.pone.0007506 19841742 PMC2759555

[B76] Gomarasca M. Banfi G. Lombardi G. (2020). Myokines: The endocrine coupling of skeletal muscle and bone. *Adv. Clin. Chem.* 94 155–218. 10.1016/bs.acc.2019.07.010 31952571

[B77] Gontier G. George C. Chaker Z. Holzenberger M. Aid S. (2015). Blocking IGF Signaling in Adult Neurons Alleviates Alzheimer’s Disease Pathology through Amyloid-beta Clearance. *J. Neurosci.* 35 11500–11513. 10.1523/JNEUROSCI.0343-15.2015 26290229 PMC6605240

[B78] Goto T. Yamaza T. Kido M. A. Tanaka T. (1998). Light- and electron-microscopic study of the distribution of axons containing substance P and the localization of neurokinin-1 receptor in bone. *Cell Tissue Res.* 293 87–93. 10.1007/s004410051100 9634600

[B79] Goulding S. R. Sullivan A. M. O’Keeffe G. W. Collins L. M. (2020). The potential of bone morphogenetic protein 2 as a neurotrophic factor for Parkinson’s disease. *Neural. Regen Res.* 15 1432–1436. 10.4103/1673-5374.274327 31997802 PMC7059567

[B80] Grasso D. Corsetti R. Lanteri P. Di Bernardo C. Colombini A. Graziani R. (2015). Bone-muscle unit activity, salivary steroid hormones profile, and physical effort over a 3-week stage race. *Scand. J. Med. Sci. Sport* 25 70–80. 10.1111/sms.12147 24433517

[B81] Gu Y. Genever P. G. Skerry T. M. Publicover S. J. (2002). The NMDA type glutamate receptors expressed by primary rat osteoblasts have the same electrophysiological characteristics as neuronal receptors. *Calcif Tissue Int.* 70 194–203. 10.1007/s00223-001-2004-z 11907717

[B82] Hanada R. Leibbrandt A. Hanada T. Kitaoka S. Furuyashiki T. Fujihara H. (2009). Central control of fever and female body temperature by RANKL/RANK. *Nature* 462 505–509. 10.1038/nature08596 19940926

[B83] Hanami K. Nakano K. Saito K. Okada Y. Yamaoka K. Kubo S. (2013). Dopamine D2-like receptor signaling suppresses human osteoclastogenesis. *Bone* 56 1–8. 10.1016/j.bone.2013.04.019 23631878

[B84] Hauschka P. V. Lian J. B. Cole D. E. Gundberg C. M. (1989). Osteocalcin and matrix Gla protein: vitamin K-dependent proteins in bone. *Physiol Rev.* 69 990–1047. 2664828 10.1152/physrev.1989.69.3.990

[B85] Hayashi M. Nakashima T. Yoshimura N. Okamoto K. Tanaka S. Takayanagi H. (2019). Autoregulation of Osteocyte Sema3A Orchestrates Estrogen Action and Counteracts Bone Aging. *Cell Metab.* 29 627–37e5. 10.1016/j.cmet.2018.12.021 30661929

[B86] Heisz J. J. Clark I. B. Bonin K. Paolucci E. M. Michalski B. Becker S. (2017). The Effects of Physical Exercise and Cognitive Training on Memory and Neurotrophic Factors. *J. Cogn. Neurosci.* 29 1895–1907. 10.1162/jocn_a_01164 28699808

[B87] Heisz J. J. Vandermorris S. Wu J. McIntosh A. R. Ryan J. D. (2015). Age differences in the association of physical activity, sociocognitive engagement, and TV viewing on face memory. *Health psychol.* 83–88. 10.1037/hea0000046 24467255

[B88] Hensel N. Schon A. Konen T. Lubben V. Forthmann B. Baron O. (2016). Fibroblast growth factor 23 signaling in hippocampal cells: impact on neuronal morphology and synaptic density. *J. Neurochem.* 137 756–769. 10.1111/jnc.13585 26896818

[B89] Hill H. S. Grams J. Walton R. G. Liu J. Moellering D. R. Garvey W. T. (2014). Carboxylated and Uncarboxylated Forms of Osteocalcin Directly Modulate the Glucose Transport System and Inflammation in Adipocytes. *Hormone. Metab. Res.* 46 341–347. 10.1055/s-0034-1368709 24554534 PMC5349189

[B90] Hillman C. H. Erickson K. I. Kramer A. F. (2008). Be smart, exercise your heart: exercise effects on brain and cognition. *Nat. Rev. Neurosci.* 9 58–65. 10.1038/nrn2298 18094706

[B91] Hinoi E. Fujimori S. Takarada T. Taniura H. Yoneda Y. (2002). Facilitation of glutamate release by ionotropic glutamate receptors in osteoblasts. *Biochem. Biophys. Res. Commun.* 297 452–458. 10.1016/s0006-291x(02)02223-4 12270113

[B92] Ho M. L. Tsai T. N. Chang J. K. Shao T. S. Jeng Y. R. Hsu C. (2005). Down-regulation of N-methyl D-aspartate receptor in rat-modeled disuse osteopenia. *Osteoporos. Int.* 16 1780–1788. 10.1007/s00198-005-1928-y 15997422

[B93] Holguin N. Brodt M. D. Silva M. J. (2016). Activation of Wnt Signaling by Mechanical Loading Is Impaired in the Bone of Old Mice. *J. Bone Miner. Res.* 31 2215–2226. 10.1002/jbmr.2900 27357062 PMC5397287

[B94] Hsuchou H. Pan W. Kastin A. J. (2013). Fibroblast growth factor 19 entry into brain. *Fluids Barriers CNS* 10:32. 10.1186/2045-8118-10-32 24176017 PMC3818657

[B95] Huang T. Larsen K. T. Ried-Larsen M. Moller N. C. Andersen L. B. (2014). The effects of physical activity and exercise on brain-derived neurotrophic factor in healthy humans: A review. *Scand J. Med. Sci. Sport* 24 1–10. 10.1111/sms.12069 23600729

[B96] Huang Y. Liu L. Liu A. (2018). Dickkopf-1: Current knowledge and related diseases. *Life Sci.* 209 249–254. 10.1016/j.lfs.2018.08.019 30102902

[B97] Igwe J. C. Jiang X. Paic F. Ma L. Adams D. J. Baldock P. A. (2009). Neuropeptide Y is expressed by osteocytes and can inhibit osteoblastic activity. *J. Cell Biochem.* 108 621–630. 10.1002/jcb.22294 19670271 PMC2754602

[B98] Jovanovic V. M. Salti A. Tilleman H. Zega K. Jukic M. M. Zou H. (2018). BMP/SMAD Pathway Promotes Neurogenesis of Midbrain Dopaminergic Neurons In Vivo and in Human Induced Pluripotent and Neural Stem Cells. *J. Neurosci.* 38 1662–1676. 10.1523/JNEUROSCI.1540-17.2018 29321139 PMC5815451

[B99] Juarranz Y. Abad C. Martinez C. Arranz A. Gutierrez-Canas I. Rosignoli F. (2005). Protective effect of vasoactive intestinal peptide on bone destruction in the collagen-induced arthritis model of rheumatoid arthritis. *Arthritis. Res. Ther.* 7 R1034–R1045. 10.1186/ar1779 16207319 PMC1257432

[B100] Kajimura D. Lee H. W. Riley K. J. Arteaga-Solis E. Ferron M. Zhou B. (2013). Adiponectin regulates bone mass via opposite central and peripheral mechanisms through FoxO1. *Cell Metabol.* 17 901–915. 10.1016/j.cmet.2013.04.009 23684624 PMC3679303

[B101] Kaleta B. (2019). The role of osteopontin in kidney diseases. *Inflamm. Res.* 68 93–102. 10.1007/s00011-018-1200-5 30456594

[B102] Kanazawa I. (2015). Osteocalcin as a hormone regulating glucose metabolism. *World J. Diabetes* 6 1345–1354. 10.4239/wjd.v6.i18.1345 26722618 PMC4689779

[B103] Katsimpardi L. Litterman N. K. Schein P. A. Miller C. M. Loffredo F. S. Wojtkiewicz G. R. (2014). Vascular and neurogenic rejuvenation of the aging mouse brain by young systemic factors. *Science* 344 630–634. 10.1126/science.1251141 24797482 PMC4123747

[B104] Kauschke V. Gebert A. Calin M. Eckert J. Scheich S. Heiss C. (2018). Effects of new beta-type Ti-40Nb implant materials, brain-derived neurotrophic factor, acetylcholine and nicotine on human mesenchymal stem cells of osteoporotic and non osteoporotic donors. *PLoS One* 13:e0193468. 10.1371/journal.pone.0193468 29489907 PMC5873971

[B105] Kegelman C. D. Coulombe J. C. Jordan K. M. Horan D. J. Qin L. Robling A. G. (2020). YAP and TAZ Mediate Osteocyte Perilacunar/Canalicular Remodeling. *J. Bone Miner Res.* 35 196–210. 10.1002/jbmr.3876 31610061 PMC7066596

[B106] Kegelman C. D. Mason D. E. Dawahare J. H. Horan D. J. Vigil G. D. Howard S. S. (2018). Skeletal cell YAP and TAZ combinatorially promote bone development. *FASEB J.* 32 2706–2721. 10.1096/fj.201700872R 29401582 PMC5901392

[B107] Khosla S. Drake M. T. Volkman T. L. Thicke B. S. Achenbach S. J. Atkinson E. J. (2018). Sympathetic beta1-adrenergic signaling contributes to regulation of human bone metabolism. *J. Clin. Invest.* 128 4832–4842. 10.1172/JCI122151 30153111 PMC6205387

[B108] Khrimian L. Obri A. Ramos-Brossier M. Rousseaud A. Moriceau S. Nicot A. S. (2017). Gpr158 mediates osteocalcin’s regulation of cognition. *J. Exp. Med.* 214 2859–2873. 10.1084/jem.20171320 28851741 PMC5626410

[B109] Kim J. G. Sun B. H. Dietrich M. O. Koch M. Yao G. Q. Diano S. (2015). AgRP Neurons Regulate Bone Mass. *Cell Rep.* 13 8–14. 10.1016/j.celrep.2015.08.070 26411686 PMC5868421

[B110] Kim T. H. Chang J. S. Park K. S. Park J. Kim N. Lee J. I. (2017). Effects of exercise training on circulating levels of Dickkpof-1 and secreted frizzled-related protein-1 in breast cancer survivors: A pilot single-blind randomized controlled trial. *PLoS One* 12:e0171771. 10.1371/journal.pone.0171771 28178355 PMC5298304

[B111] Kodama D. Hirai T. Kondo H. Hamamura K. Togari A. (2017). Bidirectional communication between sensory neurons and osteoblasts in an in vitro coculture system. *FEBS Lett.* 591 527–539. 10.1002/1873-3468.12561 28094440

[B112] Kunert S. K. Hartmann H. Haffner D. Leifheit-Nestler M. (2017). Klotho and fibroblast growth factor 23 in cerebrospinal fluid in children. *J. Bone Miner. Metab.* 35 215–226. 10.1007/s00774-016-0746-y 27017221

[B113] Lanteri P. Lombardi G. Colombini A. Grasso D. Banfi G. (2012). Stability of osteopontin in plasma and serum. *Clin. Chem. Lab. Med.* 50 1979–1984. 10.1515/cclm-2012-0177 22718644

[B114] Laszczyk A. M. Nettles D. Pollock T. A. Fox S. Garcia M. L. Wang J. (2019). FGF-23 Deficiency Impairs Hippocampal-Dependent Cognitive Function. *eNeuro* 6:2019. 10.1523/ENEURO.0469-18.2019 30911673 PMC6430630

[B115] Laviola L. Natalicchio A. Perrini S. Giorgino F. (2008). Abnormalities of IGF-I signaling in the pathogenesis of diseases of the bone, brain, and fetoplacental unit in humans. *Am. J. Physiol. Endocrinol. Metabm.* 295 E991–E999. 10.1152/ajpendo.90452.2008 18713961

[B116] Lee D. J. Tseng H. C. Wong S. W. Wang Z. Deng M. Ko C. C. (2015). Dopaminergic effects on in vitro osteogenesis. *Bone Res* 3 15020. 10.1038/boneres.2015.20 26558139 PMC4639997

[B117] Lee N. J. Nguyen A. D. Enriquez R. F. Luzuriaga J. Bensellam M. Laybutt R. (2015). NPY signalling in early osteoblasts controls glucose homeostasis. *Mol. Metab.* 4 164–174. 10.1016/j.molmet.2014.12.010 25737952 PMC4338316

[B118] Lee T. H. Formolo D. A. Kong T. Lau S. W. Ho C. S. Leung R. Y. H. (2019). Potential exerkines for physical exercise-elicited pro-cognitive effects: Insight from clinical and animal research. *Int. Rev. Neurobiol.* 147 361–395. 10.1016/bs.irn.2019.06.002 31607361

[B119] Lewis K. J. Frikha-Benayed D. Louie J. Stephen S. Spray D. C. Thi M. M. (2017). Osteocyte calcium signals encode strain magnitude and loading frequency in vivo. *Proc. Nat. Acad. Sci. USA* 114 11775–11780. 10.1073/pnas.1707863114 29078317 PMC5676898

[B120] Li D. J. Fu H. Zhao T. Ni M. Shen F. M. (2016). Exercise-stimulated FGF23 promotes exercise performance via controlling the excess reactive oxygen species production and enhancing mitochondrial function in skeletal muscle. *Metabol. Clin. Exp.* 65 747–756. 10.1016/j.metabol.2016.02.009 27085781

[B121] Li J. Sarosi I. Cattley R. C. Pretorius J. Asuncion F. Grisanti M. (2006). Dkk1-mediated inhibition of Wnt signaling in bone results in osteopenia. *Bone* 39 754–766. 10.1016/j.bone.2006.03.017 16730481

[B122] Li X. Han L. Nookaew I. Mannen E. Silva M. J. Almeida M. (2019). Stimulation of Piezo1 by mechanical signals promotes bone anabolism. *eLife* 8:49631. 10.7554/eLife.49631 31588901 PMC6779475

[B123] Li X. Zhang Y. Kang H. Liu W. Liu P. Zhang J. (2005). Sclerostin binds to LRP5/6 and antagonizes canonical Wnt signaling. *J. Biol. Chem.* 280 19883–19887. 10.1074/jbc.M413274200 15778503

[B124] Liang H. Zeng Y. Feng Y. Wu H. Gong P. Yao Q. (2018). Selective beta2-adrenoreceptor signaling regulates osteoclastogenesis via modulating RANKL production and neuropeptides expression in osteocytic MLO-Y4 cells. *J. Cell Biochem.* 2018:27998. 10.1002/jcb.27998 30387222

[B125] Libro R. Bramanti P. Mazzon E. (2016). The role of the Wnt canonical signaling in neurodegenerative diseases. *Life Sci.* 158 78–88. 10.1016/j.lfs.2016.06.024 27370940

[B126] Liu S. Zhou J. Tang W. Jiang X. Rowe D. W. Quarles L. D. (2006). Pathogenic role of Fgf23 in Hyp mice. *Am. J. Physiol. Endocrinol. Metab.* 291 E38–E49. 10.1152/ajpendo.00008.2006 16449303

[B127] Liu W. Zhang X. (2015). Receptor activator of nuclear factor-kappaB ligand (RANKL)/RANK/osteoprotegerin system in bone and other tissues (review). *Mol. Med. Rep.* 11 3212–3218. 10.3892/mmr.2015.3152 25572286

[B128] Lombardi G. Barbaro M. Locatelli M. Banfi G. (2017). Novel bone metabolism-associated hormones: the importance of the pre-analytical phase for understanding their physiological roles. *Endocrine* 56 460–484. 10.1007/s12020-017-1239-z 28181144

[B129] Lombardi G. Corsetti R. Lanteri P. Grasso D. Vianello E. Marazzi M. G. (2014). Reciprocal regulation of calcium-/phosphate-regulating hormones in cyclists during the Giro d’Italia 3-week stage race. *Scand J. Med. Sci. Sport* 24 779–787. 10.1111/sms.12080 23647316

[B130] Lombardi G. Lanteri P. Colombini A. Mariotti M. Banfi G. (2012a). Sclerostin concentrations in athletes: role of load and gender. *J. Biol. Regul. Homeost Agents* 26 157–163. Epub 2012/04/06. 18 [pii]. 22475109

[B131] Lombardi G. Lanteri P. Graziani R. Colombini A. Banfi G. Corsetti R. (2012b). Bone and energy metabolism parameters in professional cyclists during the Giro d’Italia 3-weeks stage race. *PLoS One* 7:e42077. 10.1371/journal.pone.0042077 22848709 PMC3407078

[B132] Lombardi G. Perego S. Luzi L. Banfi G. A. (2015). four-season molecule: osteocalcin. Updates in its physiological roles. *Endocrine* 48 394–404. 25158976 10.1007/s12020-014-0401-0

[B133] Lombardi G. Sanchis-Gomar F. Perego S. Sansoni V. Banfi G. (2016). Implications of exercise-induced adipo-myokines in bone metabolism. *Endocrine* 54 284–305. 26718191 10.1007/s12020-015-0834-0

[B134] Loskutova N. Honea R. A. Vidoni, Brooks W. M. Burns J. M. (2009). Bone density and brain atrophy in early Alzheimer’s disease. *J. Alzheimer. Dis.* 18 777–785. 10.3233/JAD-2009-1185 19661621 PMC2842449

[B135] Lourenco M. V. Frozza R. L. de Freitas G. B. Zhang H. Kincheski G. C. Ribeiro F. C. (2019). Exercise-linked FNDC5/irisin rescues synaptic plasticity and memory defects in Alzheimer’s models. *Nat. Med.* 25 165–175. 10.1038/s41591-018-0275-4 30617325 PMC6327967

[B136] Ma W. Zhang X. Shi S. Zhang Y. (2013). Neuropeptides stimulate human osteoblast activity and promote gap junctional intercellular communication. *Neuropeptides* 47 179–186. 10.1016/j.npep.2012.12.002 23726661

[B137] Ma Y. Li X. Fu J. Li Y. Gao L. Yang L. (2014). Acetylcholine affects osteocytic MLO-Y4 cells via acetylcholine receptors. *Mol. Cell Endocrinol.* 384 155–164. 10.1016/j.mce.2014.01.021 24508663

[B138] Maass A. Duzel S. Brigadski T. Goerke M. Becke A. Sobieray U. (2016). Relationships of peripheral IGF-1, VEGF and BDNF levels to exercise-related changes in memory, hippocampal perfusion and volumes in older adults. *NeuroImage* 131 142–154. 10.1016/j.neuroimage.2015.10.084 26545456

[B139] Mach D. B. Rogers S. D. Sabino M. C. Luger N. M. Schwei M. J. Pomonis J. D. (2002). Origins of skeletal pain: sensory and sympathetic innervation of the mouse femur. *Neurosci* 113 155–166. 10.1016/s0306-4522(02)00165-3 12123694

[B140] Maetzler W. Berg D. Schalamberidze N. Melms A. Schott K. Mueller J. C. (2007). Osteopontin is elevated in Parkinson’s disease and its absence leads to reduced neurodegeneration in the MPTP model. *Neurobiol. Dis.* 25 473–482. 10.1016/j.nbd.2006.10.020 17188882

[B141] Malashkevich V. N. Almo S. C. Dowd T. L. X. - (2013). ray crystal structure of bovine 3 Glu-osteocalcin. *Biochemistry* 52 8387–8392. 10.1021/bi4010254 24138653 PMC4517604

[B142] Mandl P. Hayer S. Karonitsch T. Scholze P. Gyori D. Sykoutri D. (2016). Nicotinic acetylcholine receptors modulate osteoclastogenesis. *Arthritis Res. Ther.* 18:63. 10.1186/s13075-016-0961-x 26970742 PMC4789270

[B143] Martinez Munoz I. Y. Camarillo Romero Garduno Garcia J. J. (2018). Irisin a Novel Metabolic Biomarker: Present Knowledge and Future Directions. *Int. J. Endocrinol.* 2018:7816806. 10.1155/2018/7816806 30402097 PMC6198573

[B144] Mason D. J. Suva L. J. Genever P. G. Patton A. J. Steuckle S. Hillam R. A. (1997). Mechanically regulated expression of a neural glutamate transporter in bone: a role for excitatory amino acids as osteotropic agents? *Bone* 20 199–205. 10.1016/s8756-3282(96)00386-9 9071469

[B145] Mastroiacovo F. Busceti C. L. Biagioni F. Moyanova S. G. Meisler M. H. Battaglia G. (2009). Induction of the Wnt antagonist, Dickkopf-1, contributes to the development of neuronal death in models of brain focal ischemia. *J. Cereb. Blood Flow Metab.* 29 264–276. 10.1038/jcbfm.2008.111 18827832

[B146] Mattson M. P. (2008). Glutamate and neurotrophic factors in neuronal plasticity and disease. *Ann. NY Acad. Sci.* 1144 97–112. 10.1196/annals.1418.005 19076369 PMC2614307

[B147] Mera P. Laue K. Ferron M. Confavreux C. Wei J. Galan-Diez M. (2016). Osteocalcin Signaling in Myofibers Is Necessary and Sufficient for Optimum Adaptation to Exercise. *Cell Metab.* 23 1078–1092. 10.1016/j.cmet.2016.05.004 27304508 PMC4910629

[B148] Millar S. A. Anderson S. I. O’Sullivan S. E. (2019). Osteokines and the vasculature: a review of the in vitro effects of osteocalcin, fibroblast growth factor-23 and lipocalin-2. *PeerJ.* 7:e7139. 10.7717/peerj.7139 31372314 PMC6660824

[B149] Mohammad Rahimi G. R. Bijeh N. Rashidlamir A. (2020). Effects of exercise training on serum preptin, undercarboxylated osteocalcin and high molecular weight adiponectin in adults with metabolic syndrome. *Exp. Physiol.* 105 449–459. 10.1113/EP088036 31869474

[B150] Morimoto R. Uehara S. Yatsushiro S. Juge N. Hua Z. Senoh S. (2006). Secretion of L-glutamate from osteoclasts through transcytosis. *EMBO J.* 25 4175–4186. 10.1038/sj.emboj.7601317 16957773 PMC1570443

[B151] Morinobu M. Ishijima M. Rittling S. R. Tsuji K. Yamamoto H. Nifuji A. (2003). Osteopontin expression in osteoblasts and osteocytes during bone formation under mechanical stress in the calvarial suture in vivo. *J. Bone Miner. Res.* 18 1706–1715. 10.1359/jbmr.2003.18.9.1706 12968681

[B152] Morrell A. E. Brown G. N. Robinson S. T. Sattler R. L. Baik A. D. Zhen G. (2018). Mechanically induced Ca(2+) oscillations in osteocytes release extracellular vesicles and enhance bone formation. *Bone Res.* 6:6. 10.1038/s41413-018-0007-x 29581909 PMC5859015

[B153] Mosialou I. Shikhel S. Liu J. M. Maurizi A. Luo N. He Z. (2017). MC4R-dependent suppression of appetite by bone-derived lipocalin 2. *Nature* 543 385–390. 10.1038/nature21697 28273060 PMC5975642

[B154] Mueller W. M. Gregoire F. M. Stanhope K. L. Mobbs C. V. Mizuno T. M. Warden C. H. (1998). Evidence that glucose metabolism regulates leptin secretion from cultured rat adipocytes. *Endocrinology* 139 551–558. 10.1210/endo.139.2.5716 9449624

[B155] Mukohyama H. Ransjo M. Taniguchi H. Ohyama T. Lerner U. H. (2000). The inhibitory effects of vasoactive intestinal peptide and pituitary adenylate cyclase-activating polypeptide on osteoclast formation are associated with upregulation of osteoprotegerin and downregulation of RANKL and RANK. *Biochem. Biophys. Res. Commun.* 271 158–163. 10.1006/bbrc.2000.2599 10777696

[B156] Niimura M. Sato T. Enoki Y. Okubo M. Kokabu S. Takeda S. (2016). Semaphorin 3A Promotes Dendrite Elongation of Osteocytes in Association with Down-regulation of CDK6. *In Vivo* 30 231–236. 27107080

[B157] Olson A. K. Eadie B. D. Ernst C. Christie B. R. (2006). Environmental enrichment and voluntary exercise massively increase neurogenesis in the adult hippocampus via dissociable pathways. *Hippocampus* 16 250–260. 10.1002/hipo.20157 16411242

[B158] Otani T. Mizokami A. Hayashi Y. Gao J. Mori Y. Nakamura S. (2015). Signaling pathway for adiponectin expression in adipocytes by osteocalcin. *Cell Signal.* 27 532–544. 10.1016/j.cellsig.2014.12.018 25562427

[B159] Oury F. Khrimian L. Denny C. A. Gardin A. Chamouni A. Goeden N. (2013). Maternal and offspring pools of osteocalcin influence brain development and functions. *Cell* 155 228–241. 10.1016/j.cell.2013.08.042 24074871 PMC3864001

[B160] Oury F. Sumara G. Sumara O. Ferron M. Chang H. Smith C. E. (2011). Endocrine regulation of male fertility by the skeleton. *Cell* 144 796–809. 10.1016/j.cell.2011.02.004 21333348 PMC3052787

[B161] Owen R. Reilly G. C. (2018). In vitro Models of Bone Remodelling and Associated Disorders. *Front. Bioeng. Biotechnol.* 6:134. 10.3389/fbioe.2018.00134 30364287 PMC6193121

[B162] Parfitt A. M. (2000). The mechanism of coupling: a role for the vasculature. *Bone* 26 319–323. 10.1016/S8756-3282(00)80937-0 10787232

[B163] Park K. R. Kim E. C. Hong J. T. Yun H. M. (2018). Dysregulation of 5-hydroxytryptamine 6 receptor accelerates maturation of bone-resorbing osteoclasts and induces bone loss. *Theranostics* 8 3087–3098. 10.7150/thno.24426 29896304 PMC5996355

[B164] Patti A. Gennari L. Merlotti D. Dotta F. Nuti R. (2013). Endocrine actions of osteocalcin. *Int. J. Endocrinol.* 2013:846480. 10.1155/2013/846480 23737779 PMC3657394

[B165] Pinzone J. J. Hall B. M. Thudi N. K. Vonau M. Qiang Y. W. Rosol T. J. (2009). The role of Dickkopf-1 in bone development, homeostasis, and disease. *Blood* 113 517–525. 10.1182/blood-2008-03-145169 18687985 PMC2628360

[B166] Plotkin L. I. Mathov I. Aguirre J. I. Parfitt A. M. Manolagas S. C. Bellido T. (2005). Mechanical stimulation prevents osteocyte apoptosis: requirement of integrins, Src kinases, and ERKs. *Am. J. Physiol. Cell Physiol.* 289 C633–C643. 10.1152/ajpcell.00278.2004 15872009

[B167] Poser J. W. Esch F. S. Ling N. C. Price P. A. (1980). Isolation and sequence of the vitamin K-dependent protein from human bone. Undercarboxylation of the first glutamic acid residue. *J. Biol. Chem.* 255 8685–8691. Epub 1980/09/25., 6967872

[B168] Puig J. Blasco G. Daunis-i-Estadella J. Moreno M. Molina X. Alberich-Bayarri A. (2016). Lower serum osteocalcin concentrations are associated with brain microstructural changes and worse cognitive performance. *Clin. Endocrinol.* 84 756–763. 10.1111/cen.12954 26406918

[B169] Purro S. A. Dickins E. M. Salinas P. C. (2012). The secreted Wnt antagonist Dickkopf-1 is required for amyloid beta-mediated synaptic loss. *J. Neurosci.* 32 3492–3498. 10.1523/JNEUROSCI.4562-11.2012 22399772 PMC6621056

[B170] Qin L. Liu W. Cao H. Xiao G. (2020). Molecular mechanosensors in osteocytes. *Bone Res.* 8:23. 10.1038/s41413-020-0099-y 32550039 PMC7280204

[B171] Reinholt F. P. Hultenby K. Oldberg A. Heinegard D. (1990). Osteopontin–a possible anchor of osteoclasts to bone. *Proc. Nat. Acad. Sci. USA* 87 4473–4475. 10.1073/pnas.87.12.4473 1693772 PMC54137

[B172] Robinson L. J. Tourkova I. Wang Y. Sharrow A. C. Landau M. S. Yaroslavskiy B. B. (2010). FSH-receptor isoforms and FSH-dependent gene transcription in human monocytes and osteoclasts. *Biochem. Biophys. Res. Commun.* 394 12–17. 10.1016/j.bbrc.2010.02.112 20171950 PMC2856932

[B173] Robling A. G. Bonewald L. F. (2020). The Osteocyte: New Insights. *Ann. Rev. Physiol.* 82 485–506. 10.1146/annurev-physiol-021119-034332 32040934 PMC8274561

[B174] Robling A. G. Turner C. H. (2009). Mechanical signaling for bone modeling and remodeling. *Crit. Rev. Eukariot. Gene Expr.* 19 319–338. 10.1615/critreveukargeneexpr.v19.i4.50 19817708 PMC3743123

[B175] Robling A. G. Niziolek P. J. Baldridge L. A. Condon K. W. Allen M. R. Alam I. (2008). Mechanical stimulation of bone in vivo reduces osteocyte expression of Sost/sclerostin. *J. Biol. Chem.* 283 5866–5875. 10.1074/jbc.M705092200 18089564

[B176] Roos P. M. (2014). Osteoporosis in neurodegeneration. *J. Trace Elem. Med. Biol.* 28 418–421. 10.1016/j.jtemb.2014.08.010 25220531

[B177] Rosen C. J. (2004). Insulin-like growth factor I and bone mineral density: experience from animal models and human observational studies. *BestPract. Res. Clin. Endcorinol. Metab.* 18 423–435. 10.1016/j.beem.2004.02.007 15261847

[B178] Rosi M. C. Luccarini I. Grossi C. Fiorentini A. Spillantini M. G. Prisco A. (2010). Increased Dickkopf-1 expression in transgenic mouse models of neurodegenerative disease. *J. Neurochem.* 112 1539–1551. 10.1111/j.1471-4159.2009.06566.x 20050968

[B179] Ross R. D. Shah R. C. Leurgans S. Bottiglieri T. Wilson R. S. Sumner D. R. (2018). Circulating Dkk1 and TRAIL Are Associated With Cognitive Decline in Community-Dwelling, Older Adults With Cognitive Concerns. *J. Gerontol. A Biol. Sci. Med. Sci.* 73 1688–1694. 10.1093/gerona/glx252 29432613 PMC6230213

[B180] Roth J. A. Kim B. G. Lin W. L. Cho M. I. (1999). Melatonin promotes osteoblast differentiation and bone formation. *J. Biol. Chem.* 274 22041–22047. 10.1074/jbc.274.31.22041 10419530

[B181] Rucci N. Capulli M. Piperni S. G. Cappariello A. Lau P. Frings-Meuthen P. (2015). Lipocalin 2: a new mechanoresponding gene regulating bone homeostasis. *J. Bone Miner Res.* 30 357–368. 10.1002/jbmr.2341 25112732

[B182] Salter D. M. Millward-Sadler S. J. Nuki G. Wright M. O. (2001). Integrin-interleukin-4 mechanotransduction pathways in human chondrocytes. *Clin. Orthopaed. Relat. Res.* 391 S49–S60. 10.1097/00003086-200110001-00006 11603724

[B183] Sampath T. K. Simic P. Sendak R. Draca N. Bowe A. E. O’Brien S. (2007). Thyroid-stimulating hormone restores bone volume, microarchitecture, and strength in aged ovariectomized rats. *J. Bone Miner Res.* 22 849–859. 10.1359/jbmr.070302 17352644

[B184] Sample S. J. Heaton C. M. Behan M. Bleedorn J. A. Racette M. A. Hao Z. (2014). Role of calcitonin gene-related peptide in functional adaptation of the skeleton. *PLoS One* 9:e113959. 10.1371/journal.pone.0113959 25536054 PMC4275203

[B185] Sansoni V. Vernillo G. Perego S. Barbuti A. Merati G. Schena F. (2017). Bone turnover response is linked to both acute and established metabolic changes in ultra-marathon runners. *Endocrine* 56 196–204. 10.1007/s12020-016-1012-8 27422791

[B186] Sato S. Hanada R. Kimura A. Abe T. Matsumoto T. Iwasaki M. (2007). Central control of bone remodeling by neuromedin U. *Nat. Med.* 13 1234–1240. 10.1038/nm1640 17873881

[B187] Sato T. Abe T. Chida D. Nakamoto N. Hori N. Kokabu S. (2010). Functional role of acetylcholine and the expression of cholinergic receptors and components in osteoblasts. *FEBS Lett.* 584 817–824. 10.1016/j.febslet.2010.01.001 20067796

[B188] Schreivogel S. Kuchibhotla V. Knaus P. Duda G. N. Petersen A. (2019). Load-induced osteogenic differentiation of mesenchymal stromal cells is caused by mechano-regulated autocrine signaling. *J. Tissue Eng. Regen. Med.* 13 1992–2008. 10.1002/term.2948 31359634

[B189] Sen B. Xie Z. Case N. Styner M. Rubin C. T. Rubin J. (2011). Mechanical signal influence on mesenchymal stem cell fate is enhanced by incorporation of refractory periods into the loading regimen. *J. Biomech.* 44 593–599. 10.1016/j.jbiomech.2010.11.022 21130997 PMC3042527

[B190] Seriwatanachai D. Krishnamra N. van Leeuwen J. P. (2009). Evidence for direct effects of prolactin on human osteoblasts: Inhibition of cell growth and mineralization. *J. Cell Biochem.* 107 677–685. 10.1002/jcb.22161 19365811

[B191] Seriwatanachai D. Thongchote K. Charoenphandhu N. Pandaranandaka J. Tudpor K. Teerapornpuntakit J. (2008). Prolactin directly enhances bone turnover by raising osteoblast-expressed receptor activator of nuclear factor kappaB ligand/osteoprotegerin ratio. *Bone* 42 535–546. 10.1016/j.bone.2007.11.008 18166509

[B192] Shan C. Ghosh A. Guo X. Z. Wang S. M. Hou Y. F. Li S. T. (2019). Roles for osteocalcin in brain signalling: implications in cognition- and motor-related disorders. *Mol. Brain* 12:23. 10.1186/s13041-019-0444-5 30909971 PMC6434857

[B193] Shcheglovitov A. Shcheglovitova O. Yazawa M. Portmann T. Shu R. Sebastiano V. (2013). SHANK3 and IGF1 restore synaptic deficits in neurons from 22q13 deletion syndrome patients. *Nature* 503 267–271. 10.1038/nature12618 24132240 PMC5559273

[B194] Shi Y. C. Lin S. Castillo L. Aljanova A. Enriquez R. F. Nguyen A. D. (2011). Peripheral-specific y2 receptor knockdown protects mice from high-fat diet-induced obesity. *Obesity* 19 2137–2148. 10.1038/oby.2011.99 21546930

[B195] Shi Y. C. Lin S. Wong I. P. Baldock P. A. Aljanova A. Enriquez R. F. (2010). NPY neuron-specific Y2 receptors regulate adipose tissue and trabecular bone but not cortical bone homeostasis in mice. *PLoS One* 5:e11361. 10.1371/journal.pone.0011361 20613867 PMC2894044

[B196] Shi Z. Madden C. J. Brooks V. L. (2017). Arcuate neuropeptide Y inhibits sympathetic nerve activity via multiple neuropathways. *J. Clin. Invest.* 127 2868–2880. 10.1172/JCI92008 28628036 PMC5490747

[B197] Simioni C. Zauli G. Martelli A. M. Vitale M. Sacchetti G. Gonelli A. (2018). Oxidative stress: role of physical exercise and antioxidant nutraceuticals in adulthood and aging. *Oncotarget* 9 17181–17198. 10.18632/oncotarget.24729 29682215 PMC5908316

[B198] Simonet W. S. Lacey D. L. Dunstan C. R. Kelley M. Chang M. S. Luthy R. (1997). Osteoprotegerin: a novel secreted protein involved in the regulation of bone density. *Cell* 89 309–319. 10.1016/s0092-8674(00)80209-3 9108485

[B199] Singh A. Gill G. Kaur H. Amhmed M. Jakhu H. (2018). Role of osteopontin in bone remodeling and orthodontic tooth movement: a review. *Progr. Orthodont* 19:18. 10.1186/s40510-018-0216-2 29938297 PMC6015792

[B200] Song J. Kim O. Y. (2018). Perspectives in Lipocalin-2: Emerging Biomarker for Medical Diagnosis and Prognosis for Alzheimer’s Disease. *Clin. Nutr. Res.* 7 1–10. 10.7762/cnr.2018.7.1.1 29423384 PMC5796918

[B201] Spatz J. M. Ellman R. Cloutier A. M. Louis L. van Vliet M. Suva L. J. (2013). Sclerostin antibody inhibits skeletal deterioration due to reduced mechanical loading. *J. Bone Miner Res.* 28 865–874. 10.1002/jbmr.1807 23109229 PMC4076162

[B202] Spatz J. M. Wein M. N. Gooi J. H. Qu Y. Garr J. L. Liu S. (2015). The Wnt Inhibitor Sclerostin Is Up-regulated by Mechanical Unloading in Osteocytes in Vitro. *J. Biol. Chem.* 290 16744–16758. 10.1074/jbc.M114.628313 25953900 PMC4505423

[B203] Steiner J. L. Murphy E. A. McClellan J. L. Carmichael M. D. Davis J. M. (2011). Exercise training increases mitochondrial biogenesis in the brain. *J. Appl. Physiol.* 111 1066–1071. 10.1152/japplphysiol.00343.2011 21817111

[B204] Suda T. Takahashi N. Udagawa N. Jimi E. Gillespie M. T. Martin T. J. (1999). Modulation of osteoclast differentiation and function by the new members of the tumor necrosis factor receptor and ligand families. *Endocrine Rev.* 20 345–357. 10.1210/edrv.20.3.0367 10368775

[B205] Sun L. Peng Y. Sharrow A. C. Iqbal J. Zhang Z. Papachristou D. J. (2006). FSH directly regulates bone mass. *Cell* 125 247–260. 10.1016/j.cell.2006.01.051 16630814

[B206] Sun L. Tamma R. Yuen T. Colaianni G. Ji Y. Cuscito C. (2016). Functions of vasopressin and oxytocin in bone mass regulation. *Proc. Nat. Acad. Sci. USA* 113 164–169. 10.1073/pnas.1523762113 26699482 PMC4711832

[B207] Sun Y. Yin X. S. Guo H. Han R. K. He R. D. Chi L. J. (2013). Elevated osteopontin levels in mild cognitive impairment and Alzheimer’s disease. *Mediat. Inflamm.* 2013:615745. 10.1155/2013/615745 23576854 PMC3612435

[B208] Takeda S. Elefteriou F. Levasseur R. Liu X. Zhao L. Parker K. L. (2002). Leptin regulates bone formation via the sympathetic nervous system. *Cell* 111 305–317. 12419242 10.1016/s0092-8674(02)01049-8

[B209] Tamma R. Sun L. Cuscito C. Lu P. Corcelli M. Li J. (2013). Regulation of bone remodeling by vasopressin explains the bone loss in hyponatremia. *Proc. Nat. Acad. Sci. USA* 110 18644–18649. 10.1073/pnas.1318257110 24167258 PMC3831977

[B210] Tan Z. S. Seshadri S. Beiser A. Zhang Y. Felson D. Hannan M. T. (2005). Bone mineral density and the risk of Alzheimer disease. *Archiv. Neurol.* 62 107–111. 10.1001/archneur.62.1.107 15642856

[B211] Tapia-Rojas C. Inestrosa N. C. (2018). Loss of canonical Wnt signaling is involved in the pathogenesis of Alzheimer’s disease. *Neural. Regen Res.* 13 1705–1710. 10.4103/1673-5374.238606 30136680 PMC6128062

[B212] Taylor A. F. (2002). Osteoblastic glutamate receptor function regulates bone formation and resorption. *J. Musculoskelet. Neuronal. Interact.* 2 285–290. 15758456

[B213] Thompson W. R. Rubin C. T. Rubin J. (2012). Mechanical regulation of signaling pathways in bone. *Gene* 503 179–193. 10.1016/j.gene.2012.04.076 22575727 PMC3371109

[B214] Togari A. Arai M. Mizutani S. Mizutani S. Koshihara Y. Nagatsu T. (1997). Expression of mRNAs for neuropeptide receptors and beta-adrenergic receptors in human osteoblasts and human osteogenic sarcoma cells. *Neurosci. Lett.* 233 125–128. 10.1016/s0304-3940(97)00649-6 9350848

[B215] Tourkova I. L. Liu L. Sutjarit N. Larrouture Q. C. Luo J. Robinson L. J. (2017). Adrenocorticotropic hormone and 1,25-dihydroxyvitamin D3 enhance human osteogenesis in vitro by synergistically accelerating the expression of bone-specific genes. *Lab. Invest.* 97 1072–1083. 10.1038/labinvest.2017.62 28737765 PMC5844701

[B216] Trejo J. L. Carro E. Torres-Aleman I. (2001). Circulating insulin-like growth factor I mediates exercise-induced increases in the number of new neurons in the adult hippocampus. *J. Neurosci.* 21 1628–1634. 11222653 10.1523/JNEUROSCI.21-05-01628.2001 PMC6762955

[B217] Villeda S. A. Plambeck K. E. Middeldorp J. Castellano J. M. Mosher K. I. Luo J. (2014). Young blood reverses age-related impairments in cognitive function and synaptic plasticity in mice. *Nat. Med.* 20 659–663. 10.1038/nm.3569 24793238 PMC4224436

[B218] Wan W. Xia S. Kalionis B. Liu L. Li Y. (2014). The role of Wnt signaling in the development of Alzheimer’s disease: a potential therapeutic target? *BioMed. Res. Int.* 2014:301575. 10.1155/2014/301575 24883305 PMC4026919

[B219] Wang C. X. Ge X. Y. Wang M. Y. Ma T. Zhang Y. Lin Y. (2020). Dopamine D1 receptor-mediated activation of the ERK signaling pathway is involved in the osteogenic differentiation of bone mesenchymal stem cells. *Stem Cell Res. Ther.* 11:12. 10.1186/s13287-019-1529-x 31900224 PMC6942280

[B220] Weiss A. Attisano L. (2013). The TGFbeta superfamily signaling pathway. *Wiley Interdiscip. Rev. Dev. Biol.* 2 47–63. 10.1002/wdev.86 23799630

[B221] Wrann C. D. White J. P. Salogiannnis J. Laznik-Bogoslavski D. Wu J. Ma D. (2013). Exercise induces hippocampal BDNF through a PGC-1alpha/FNDC5 pathway. *Cell Metabol.* 18 649–659. 10.1016/j.cmet.2013.09.008 24120943 PMC3980968

[B222] Wrigley S. Arafa D. Tropea D. (2017). Insulin-Like Growth Factor 1: At the Crossroads of Brain Development and Aging. *Front. Cell Neurosci.* 11:14. 10.3389/fncel.2017.00014 28203146 PMC5285390

[B223] Xiao Z. Quarles L. D. (2015). Physiological mechanisms and therapeutic potential of bone mechanosensing. *Rev. Endocrine Metab. Disorders* 16 115–129. 10.1007/s11154-015-9313-4 26038304 PMC5079521

[B224] Yadav V. K. Oury F. Suda N. Liu Z. W. Gao X. B. Confavreux C. (2009). A serotonin-dependent mechanism explains the leptin regulation of bone mass, appetite, and energy expenditure. *Cell* 138 976–989. 10.1016/j.cell.2009.06.051 19737523 PMC2768582

[B225] Yakar S. Rosen C. J. Beamer W. G. Ackert-Bicknell C. L. Wu Y. Liu J. L. (2002). Circulating levels of IGF-1 directly regulate bone growth and density. *J. Clin. Invest.* 110 771–781. 10.1172/JCI15463 12235108 PMC151128

[B226] Yao Q. Liang H. Huang B. Xiang L. Wang T. Xiong Y. (2017). Beta-adrenergic signaling affect osteoclastogenesis via osteocytic MLO-Y4 cells’ RANKL production. *Biochem. Biophys. Res. Commun.* 488 634–640. 10.1016/j.bbrc.2016.11.011 27823934

[B227] Yasuda H. Shima N. Nakagawa N. Yamaguchi K. Kinosaki M. Mochizuki S. (1998). Osteoclast differentiation factor is a ligand for osteoprotegerin/osteoclastogenesis-inhibitory factor and is identical to TRANCE/RANKL. *Proc. Nat. Acad. Sci. USA* 95 3597–3602. 10.1073/pnas.95.7.3597 9520411 PMC19881

[B228] Yoshiko Y. Wang H. Minamizaki T. Ijuin C. Yamamoto R. Suemune S. (2007). Mineralized tissue cells are a principal source of FGF23. *Bone* 40 1565–1573. 10.1016/j.bone.2007.01.017 17350357

[B229] Yuan J. Meloni B. P. Shi T. Bonser A. Papadimitriou J. M. Mastaglia F. L. (2019). The Potential Influence of Bone-Derived Modulators on the Progression of Alzheimer’s Disease. *J. Alzheimer. Dis.* 69 59–70. 10.3233/JAD-181249 30932886

[B230] Zhang J. Fujita Y. Chang L. Pu Y. Qu Y. Wang S. (2020). Beneficial effects of anti-RANKL antibody in depression-like phenotype, inflammatory bone markers, and bone mineral density in male susceptible mice after chronic social defeat stress. *Behav. Brain. Res.* 379:112397. 10.1016/j.bbr.2019.112397 31790783

[B231] Zhang L. Su P. Xu C. Chen C. Liang A. Du K. (2010). Melatonin inhibits adipogenesis and enhances osteogenesis of human mesenchymal stem cells by suppressing PPARgamma expression and enhancing Runx2 expression. *J. Pineal. Res.* 49 364–372. 10.1111/j.1600-079X.2010.00803.x 20738756

[B232] Zhong Q. Sridhar S. Ruan L. Ding K. H. Xie D. Insogna K. (2005). Multiple melanocortin receptors are expressed in bone cells. *Bone* 36 820–831. 10.1016/j.bone.2005.01.020 15804492

